# Transcriptomic profiles of endocrine-resistant breast cancer

**DOI:** 10.1186/s12885-025-14826-1

**Published:** 2025-10-13

**Authors:** Caroline Schagerholm Stanev, Emmanouil G. Sifakis, Linnea Hases, Xinsong Chen, Cecilia Williams, Stephanie Robertson, Johan Hartman

**Affiliations:** 1 Department of Oncology-Pathology, Karolinska Institutet, Stockholm, Sweden; 2 Department of Protein Science, SciLifeLab, KTH Royal Institute of Technology, Stockholm, Sweden; 3 Department of Medicine Huddinge, Karolinska Institutet, Stockholm, Sweden; 4 Department of Clinical Pathology and Cancer Diagnostics, Karolinska University Hospital, Stockholm, Sweden

**Keywords:** Breast cancer, Endocrine resistance, Gene expression

## Abstract

**Background:**

The majority of breast cancer patients have tumors expressing estrogen receptor α (ER) and are treated with adjuvant endocrine therapy. However, nearly one-third relapse, most with retained ER expression.

**Methods:**

This study investigated patients with ER-positive and human epidermal growth factor receptor 2 (HER2)-negative primary breast cancer. Patients with ER-positive relapses within five years of ongoing endocrine therapy were defined as endocrine-resistant (*N* = 69). Patients with no disease progression after 10 years were defined as endocrine-sensitive (*N* = 77). RNA was extracted from archived tumor blocks, followed by gene expression analysis.

**Results:**

Significant differences were observed with higher tumor grades, intrinsic subtype risk scores, and upregulated cell-cycle gene sets in resistant compared to sensitive patients’ primary tumors. Metabolism-associated gene sets were upregulated, and estrogen-response gene sets downregulated in resistant patients' relapse compared to primary tumors.

**Conclusions:**

This study highlights gene sets associated with endocrine resistance and identifies transcriptomic and clinicopathological profiles that may serve as potential prognostic markers for therapy response.

**Supplementary Information:**

The online version contains supplementary material available at 10.1186/s12885-025-14826-1.

## Background

Breast cancer is the most common malignancy in females, with a global incidence of 11.6% [1]. In breast cancer diagnostics, routine biomarker immunohistochemistry (IHC) assessments of estrogen receptor α (ER/*ESR1*), human epidermal growth factor receptor 2 (HER2/*ERBB2*), and in some countries Ki67 (*MKI67*), and the progesterone receptor (PR/*PGR*) are used for classification. Together with the histological tumor grade (Nottingham Histologic Grade (NHG)) [2], these biomarkers are instrumental in predicting the prognosis and guiding adjuvant therapies [3]. Commercial gene expression tests are used to further risk stratify patients with ER-positive (ER +) and HER2-negative (HER2-) tumors based on gene expression signatures [4–9]. These multigene tests are expensive and not available in routine diagnostics worldwide. The tests are often recommended when the tumors are considered intermediate risk (e.g., NHG2) [3, 10, 11].

Approximately 75% of breast cancer patients have tumors expressing ER, and the patients are consequently offered adjuvant endocrine therapy for 5–10 years [3, 10]. Endocrine therapies significantly reduce the risk of relapse and have decreased mortality in primary breast cancer patients by 40% [12–14]. ER expression in tumor cells remains the only clinically established biomarker for predicting endocrine therapy response [2]. ER + tumors can be divided into luminal A and luminal B subtypes based on their Prediction Analysis of Microarray 50 (PAM50) gene expression signature [4, 5]. Tumors of the luminal B subtype are associated with a poor prognosis, and patients may also require chemotherapy. Luminal B tumors can also express HER2, which is amplified in 10–15% of all breast tumors and can be targeted through systemic antibody therapies. During recent years, additional targeted treatments for patients with ER + disease have emerged, in particularly inhibitors of cyclin-dependent kinase 4/6 (CDK4/6) and phosphoinositide 3-kinase (PI3K), but also protein kinase B (AKT) [15], as well as selective ER degraders for *ESR1* mutant breast cancer [16, 17].

Despite these advances, approximately 30% of patients with ER + primary tumors develop endocrine-resistant recurrences, and up to 50% have limited benefit from treatment due to either acquired or intrinsic resistance [12, 18–24]. In most patients, ER expression persists in the recurrent tumors [23], although the tumor no longer responds to endocrine therapies. Mechanistic studies have identified *ESR1* mutations in up to 40% of metastatic patients, implying constitutive activation of ER also in the absence of a ligand [25]. Nevertheless, the issue remains elusive for most patients, and additional investigations are needed to understand endocrine resistance in order to offer patients effective treatments [24, 26–28].

Our study aims to uncover transcriptional differences between tumors from patients with confirmed endocrine resistance and those responsive to therapy. By comparing the transcriptional and clinicopathological profiles of primary and recurrent tumors of endocrine-resistant patients, we strive to contribute to unraveling the underlying mechanisms driving endocrine resistance.

## Methods

### Study design

**Fig. 1:**
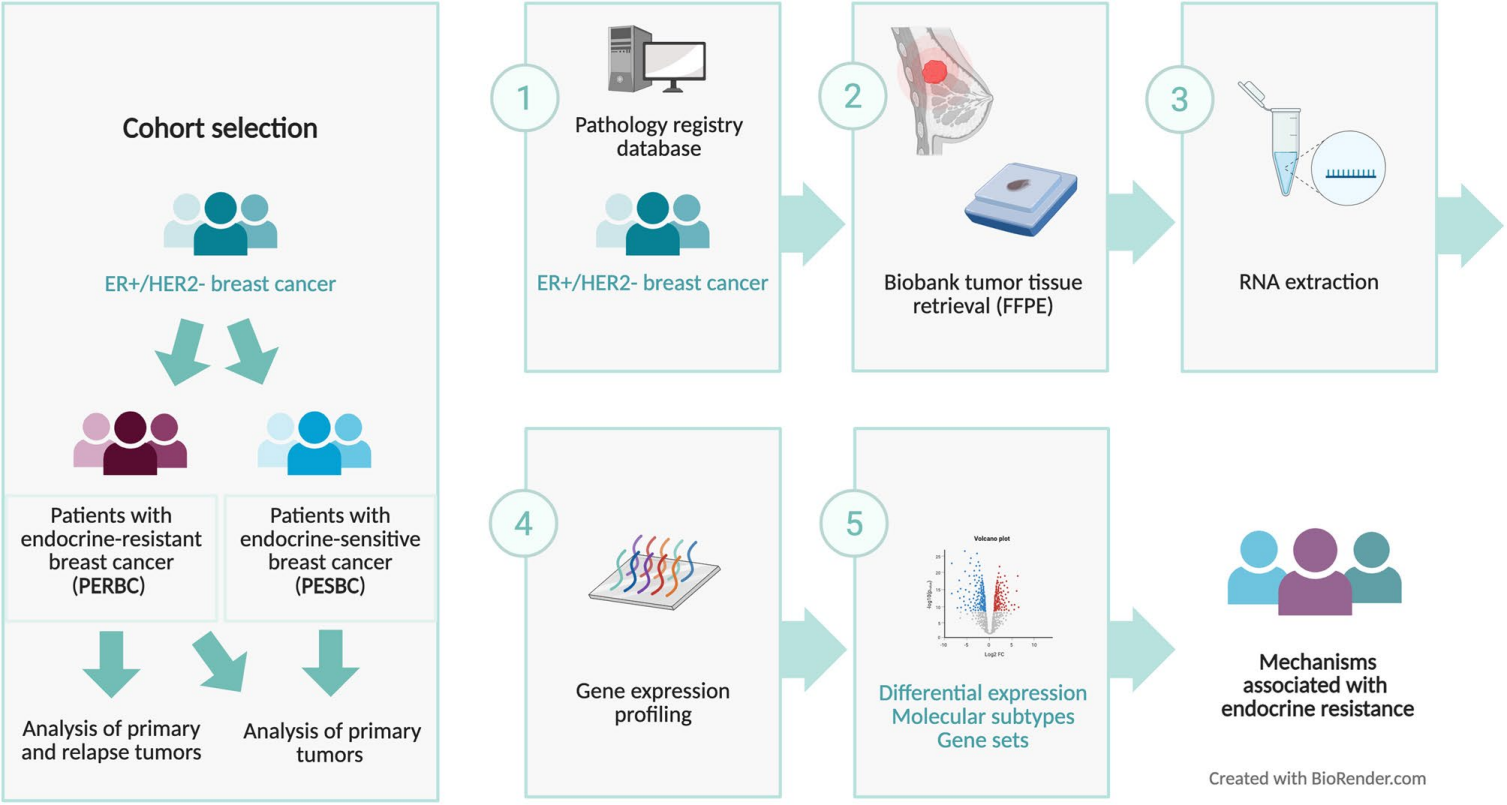
Schematic diagram of the overall study design, illustrating the study cohort selection and analytical workflow. ER = estrogen receptor, HER2 = human epidermal growth factor receptor 2, PERBC = patients with endocrine-resistant breast cancer, PESBC = patients with endocrine-sensitive breast cancer, FFPE = formalin-fixed paraffin-embedded. Created in BioRender. Karlsson, E. (2025) https://BioRender.com/h4xduu0

The study was designed as a retrospective cohort study of patients with endocrine-resistant and endocrine-sensitive breast cancer diagnosed in Stockholm, Sweden. The overall study design is visualized in a schematic diagram (Fig. 1), and the inclusion process of the entire cohort is summarized in a CONSORT diagram (Supplementary Fig. 1). Patients’ invasive tumors were identified in the pathology laboratory information system at the Karolinska University Hospital, Sweden. Patients with endocrine-resistant breast cancer (PERBC) were defined as having an ER + and HER2- primary breast tumor and a consecutive ER + relapse within five years from initial diagnosis and during ongoing endocrine therapy, diagnosed in 2009–2012. Patients with endocrine-sensitive breast cancer (PESBC) were defined as having an ER + and HER2- primary tumor without disease recurrence or progress after a follow-up period of at least 10 years, with diagnoses dating back to 2005. Resistant and sensitive patients were grouped based on tumor stage (Anatomic stage I-III, TNM classification, AJCC Breast cancer staging 7th Ed) and age at diagnosis (< 50 years or ≥ 50 years) [10]. In the absence of matched healthy breast tissue, ER⁺, HER2⁻ primary tumors from long‑term disease‑free patients (PESBC) were elected as reference group. This choice enabled focus on transcriptional alterations specifically associated with endocrine resistance, while controlling for ER status, HER2 status, tumor stage, age at diagnosis, and general tumor‑intrinsic biology.

The study initially involved two cohorts (cohort I and cohort II), collected during different periods, with variations in chemotherapy treatment and nucleic acid extraction kits (detailed in Supplementary Fig. 1 and below). The two cohorts were initially created and analyzed separately to account for potential patient characteristics and data collection differences. These preliminary analyses facilitated the evaluation of the methodology and the identification of consistent findings. Subsequently, the two cohorts were merged to enhance statistical power and enable a more comprehensive analysis of the research questions. The combined dataset, hereafter referred to as the Endoresist cohort, was then used for all subsequent analyses. The study design and reporting adhered as closely as possible to the Reporting Recommendations for Tumor Marker Prognostic Studies (REMARK) guidelines [29]. Additionally, biospecimen reporting followed the Biospecimen reporting for improved study quality (BRISQ) criteria [30], detailed in Supplementary Table 1.

### Clinical and pathological data

Clinical data were retrieved from laboratory information systems and medical records, including patient and tumor characteristics, and treatment information, available in Table 1 and Supplementary Table 2. Biomarker assessment of ER, PR, HER2, and Ki67 had been performed according to national guidelines at diagnosis and was retrospectively collected from the pathology reports. ER-positivity was assessed at ≥ 1% and PR-positivity at ≥ 10%. All included patients were diagnosed as HER2- and had not received anti-HER2 treatment. For this study, HER2 status was divided into HER2-negative/zero for all patients with HER2 IHC score 0, HER2-low for HER2 IHC scores ≥ 1 without gene amplification by HER2 in situ hybridization (ISH), and HER2-positive if gene amplified by HER2 ISH analysis (only in recurrences) [31]. Tumor-infiltrating lymphocytes (TILs) were analyzed in hematoxylin and eosin-stained sections according to international guidelines from Denkert [32] and Salgado [33] by board-certified pathologists.

**Table 1:** Clinicopathological characteristics of all primary tumors of the patients in the Endoresist cohort

	PERBC (primary tumors) N = 69	PESBC (primary tumors) N = 77	Entire cohort (PERBC & PESBC primary tumors) N = 146	P-value PERBC vs. PESBC primary tumors
**Age at diagnosis**				*p* =.96
Median: median (range), years	61 (30–88)	59 (40–81)	60 (30–88)	
**Tumor size**				*p* =.000038
Median: median (range), mm	24 (6.5–100)	17 (4–55)	20.5 (4–100)	
**Histologic subtype**				*p* =.39
Ductal	48 (69.57%)	60 (77.92%)	108 (73.97%)	
Lobular	15 (21.74%)	14 (18.18%)	29 (19.86%)	
Other	6 (8.70%)	3 (3.90%)	9 (6.16%)	
**Lymph node status**				*p* = 1
Positive	31 (44.93%)	34 (44.16%)	65 (44.52%)	
Negative	38 (55.07%)	43 (55.84%)	81 (55.48%)	
**TNM stage**				*p* =.34
Stage 1	17 (24.64%)	26 (33.77%)	43 (29.45%)	
Stage 2	37 (53.62%)	40 (51.95%)	77 (52.74%)	
Stage 3	15 (21.74%)	11 (14.29%)	26 (17.81%)	
**Tumor grade**				*p* =.013
NHG1	9 (13.04%)	13 (16.88%)	22 (15.07%)	
NHG2	32 (46.38%)	49 (63.64%)	81 (55.48%)	
NHG3	27 (39.13%)	13 (16.88%)	40 (27.40%)	
N/A	1 (1.45%)	2 (2.60%)	3 (2.05%)	
**ER, %**				*p* =.51
Median: median (range), %	90 (5–100)	90 (5–100)	90 (5–100)	
**PR, %**				*p* =.15
Median: median (range), %	50 (0–100)	50 (0–100)	50 (0–100)	
**PR status**				*p* =.0039
Negative (< 10%)	15 (21.74%)	3 (3.90%)	18 (12.33%)	
Positive (≥ 10%)	39 (56.52%)	47 (61.04%)	86 (58.90%)	
N/A	15 (21.74%)	27 (35.06%)	42 (28.77%)	
**Ki67, %**				*p* =.070
Median: median (range), %	20 (1–95)	15 (1–50)	17 (1–95)	
**HER2 status**				*p* =.14
HER2-negative/zero	42 (60.87%)	27 (35.06%)	69 (47.26%)	
HER2-low	24 (34.78%)	28 (36.36%)	52 (35.62%)	
HER2-positive	0 (0.00%)	0 (0.00%)	0 (0.00%)	
N/A	3 (4.35%)	22 (28.57%)	25 (17.12%)	
**TIL scoring**				*p* =.44
Median: median (range), %	5 (1–40)	5 (0–75)	5 (0–75)	
**Surgical procedure**				*p* =.0074
Mastectomy	33 (47.83%)	20 (25.97%)	53 (36.30%)	
Partial mastectomy	35 (50.72%)	56 (72.73%)	91 (62.33%)	
Other	0 (0.00%)	1 (1.30%)	1 (0.68%)	
N/A	1 (1.45%)	0 (0.00%)	1 (0.68%)	
**Adjuvant endocrine therapy**				*p* =.041
Tamoxifen	37 (53.62%)	49 (63.64%)	86 (58.90%)	
Aromatase inhibitor	27 (39.13%)	28 (36.36%)	55 (37.67%)	
Other	5 (7.25%)	0 (0.00%)	5 (3.42%)	
**Adjuvant chemotherapy**				*p* =.0071
Received	36 (52.17%)	23 (29.87%)	59 (40.41%)	
Not received	33 (47.83%)	54 (70.13%)	87 (59.59%)	
**Adjuvant radiotherapy**				*p* =.025
Received	45 (65.22%)	63 (81.82%)	108 (73.97%)	
Not received	24 (34.78%)	14 (18.18%)	38 (26.03%)	

PERBC = patients with endocrine-resistant breast cancer, PESBC = patients with endocrine-sensitive breast cancer, NHG = Nottingham Histologic Grade, TNM = tumor, nodal, metastasis staging, ER = estrogen receptor, PR = progesterone receptor, TIL = tumor infiltrating lymphocyte, N/A = data not available

### Tumor tissue collection

Archived formalin-fixed paraffin-embedded (FFPE) blocks and corresponding tumor glass slides were retrieved from all included patients (primary tumors, and for PERBC also paired relapse tumors) from the Stockholm Medical Biobank. From each tissue block, 10 µm tissue sections were cut by macro-dissection after markings by a pathologist to ensure invasive tumor regions on each section. Tumor tissue sections were stored in microcentrifuge tubes at 8° C.

### Included study samples

No material was available for some tumors, resulting in orphan primary or recurrent tumor samples in the PERBC. The complete cohort comprised a total of 208 samples from 146 patients consisting of 69 PERBC (with samples from 66 primary tumors and 65 relapse tumors, including 62 complete tumor pairs, 4 orphan primary tumors, and 3 orphan relapse tumors), and 77 PESBC, as seen in Supplementary Fig. 1. The relapse locations entailed 25 ipsilateral (in breast or axilla) recurrences, 20 contralateral recurrences and 24 distant metastases.

### RNA extraction and hybridization

RNA was extracted from FFPE tissue sections from all tumor samples. RNeasy FFPE Kit (50) from Qiagen, Germany, following an adjusted protocol with xylene for paraffin removal, was used for the first cohort. The RNA from the second cohort was extracted using the Qiagen AllPrep DNA/RNA FFPE Kit, also utilizing xylene for paraffin removal. AllPrep was used in the second cohort to obtain DNA for additional studies. The initial quality control (QC) of the extracted RNA was performed using the Thermo Scientific NanoDrop 2000 Spectrophotometer, measuring 260/280 nm absorbance ratio and RNA concentration. Additionally, the Agilent Bioanalyzer 2100 was employed at the core facility for RNA QC, obtaining concentration measurements, 28S/18S ratios, and RNA integrity number (RIN) values, ranging from 0.8–3.4 and concentrations of 2.43–383 ng/µl. The samples underwent microarray analysis with the Affymetrix Clariom D Human Assay at the Bioinformatics and Expression Analysis Core Facility, Karolinska Institutet [34].

### Gene expression analysis

The Transcriptome Analysis Console (TAC) version 4.0.3 (Applied Biosystems) [35] was used for the preprocessing and analysis of the microarray data. The Signal Space Transformation (SST) algorithm combined with the Robust Multi-chip Analysis (RMA) method [35] was applied to background-adjust, normalize, and summarize the raw data files. Default algorithm settings were employed for the Clariom D configuration. QC and batch effect assessments were performed using principal components analysis plots, exploratory group analyses, and the fraction of total variance, of the two cohorts, analyzed both separately and combined. The batch effects module utilizes a modified version of the ComBat function from the Surrogate Variable Analysis in R/Bioconductor [35]. Differential gene expression analysis was performed using one-way ANOVA for statistical testing, coupled with the eBayes correction. When analyzing primary tumors from PERBC and PESBC, age at diagnosis (< 50 years or ≥ 50 years) and tumor stage, used for grouping patients, were assessed as batch effects, similar to blocking factors due to the selection process. The relapse versus primary tumor analysis was performed using a repeated measures experimental design, enabling pairwise comparisons between samples from the same patients. The cohort identity (cohort I or cohort II) was considered a batch effect in the combined analyses. The sample signals were presented in log_2_ transformation for a total of 135,750 transcripts, encompassing both protein-coding and non-coding genes. The fold change (FC) in TAC is presented in linear form, with negative values representing downregulated transcripts and positive values an upregulation in the current comparison [35]. Initially, a false discovery rate (FDR) threshold of 5% was considered significant. However, due to the known quality issues associated with FFPE material and to allow for the detection of even modest changes, different thresholds for FC and p-values were evaluated, based on the volcano plots generated. Specifically, linear FC cut-offs were tested at FC >|1.2| and FC >|2|), with a p-value cut-off at *p* < 0.05. The complete gene lists from TAC were exported, and probesets were filtered based on the largest interquartile range before mapping to corresponding Entrez IDs for downstream analysis.

### Gene set enrichment analysis

Gene sets were analyzed using the Gene Set Enrichment Analysis (GSEA) method, version 4.3.3 [36, 37] to investigate differential patterns and functions associated with endocrine resistance. The Hallmark gene set, along with Kyoto Encyclopedia of Genes and Genomes (KEGG) (legacy) (https://www.kegg.jp/) and REACTOME (http://www.reactome.org/) gene sets were used in the analysis [38–41]. Normalized Enrichment Scores (NES) were employed to assess the enrichment results. An FDR q-value cut-off of < 10% was selected to reduce potential noise in the GSEA analysis [42]. The standard GSEA procedure was applied to compare primary tumors between PERBC and PESBC. For the comparison of relapse tumors to their corresponding primary tumors of PERBC, a ranked gene list was generated by multiplying the sign (positive or negative) of the linear FC by the -log_10_ of the p-value for each Entrez ID, aiming to evaluate both the statistical and biological differences between the patient’s tumor groups. The ranked list was used for preranked GSEA analysis. Primarily, the signed -log₁₀(p‑value) was employed to balance statistical confidence and regulation direction. To illustrate effect‑size–driven enrichment, GSEA ranked by log₂FC was further performed.

### External cohort

The prognostic value of the top differentially expressed transcripts and gene sets between the PERBC and PESBC primary tumors from the Endoresist cohort was evaluated using the METABRIC cohort [43], an external, well-characterized, and widely used dataset with long-term follow-up data. The top 5 upregulated and top 5 downregulated transcripts from the Endoresist cohort were selected based on FC, after filtering by *p*-value (*p* < 0.05). The top 10 gene sets from the Hallmark comparison were selected based on the NES after filtering by FDR q-value (q < 0.10). For the METABRIC cohort, gene expression microarray data (Illumina HT-12 v3 microarray) from frozen samples were downloaded from cBioPortal (https://www.cbioportal.org/), and clinicopathological and survival data were obtained from Oscar Rueda et al., 2019 [44]. The analysis included only patients with ER +/HER2- tumors, as defined by the ER.Expr and Her2.Expr variables, who received a single type of endocrine treatment (tamoxifen (TAM), aromatase inhibitors (AI), or Gonadotropin-releasing hormone agonists (GNRHA)), with or without adjuvant chemotherapy or radiotherapy (N = 989 samples).

For transcripts with duplicate IDs, the one with the highest average expression was selected. Notably, the METABRIC microarray data lacked a probe representing the gene *TRAJ14* (Entrez ID: 28741).

The Gene Set Variation Analysis (GSVA) algorithm [45], implemented in the GSVA R/Bioconductor package version 2.0.3, was used to estimate enrichment pathway scores for the Hallmark gene sets in METABRIC, as defined by the MSigDB resource [39] and assessed via the msigdbr R/Bioconductor package version 7.5.1 [46].

### Intrinsic molecular subtyping and risk of recurrence

Intrinsic molecular subtypes were estimated using the subgroup-specific gene-centering method [47], which effectively addresses the selective characteristics of ER + cohorts. Consequently, the correlation coefficient (CC) to the PAM50 centroids (basal-like, HER2-enriched, luminal A, luminal B, and normal-like) was calculated for each sample after gene-centering with the ER + subgroup percentile. Additionally, the PAM50 risk of recurrence model, the risk of recurrence (ROR)-subtype (ROR-S) were calculated as described by Parker et al. [48]. All calculations were performed within the R computing environment [49] version 4.5.0, using the BreastSubtypeR R/Bioconductor package version 1.0.0 (10.18129/B9.bioc.BreastSubtypeR).

### Chemoendocrine score

The Chemoendocrine Score (CES) was estimated as reported previously [50, 51]. Specifically, each sample's calculated CC to the luminal A and basal-like subtype centroids were used to define the CES as; CC to luminal A – CC to basal-like. CES was evaluated both as a continuous variable and as categorical groups (CES-E: endocrine-sensitive, CES-U: uncertain, CES-C: chemo-sensitive) using previously established cutoffs (CES-E vs. CES-U: 0.70; CES-U vs. CES-C: 0.30) [50, 51].

### Statistical analysis

Clinical data comparisons for the Endoresist cohort were assessed using Fisher's exact test for categorical variables and Mann–Whitney U for continuous variables. All statistical tests applied were two-sided; a p-value < 0.05 was considered statistically significant. Computations and visualizations were performed using MS Excel [52] and the R computing environment [49].

In METABRIC, survival analyses were performed with the survival R package, with Recurrence-Free Interval (RFI), as defined by the STEEP 2.0 criteria [53], serving as the primary clinical endpoint. Breast Cancer-Specific Survival (BCSS) was used as a secondary endpoint. Both endpoints were censored at 10 years.

Univariate and multivariable Cox proportional hazards (PH) regression models were applied to estimate hazard ratios (HRs) and their corresponding 95% confidence intervals (CIs). The PH assumption was tested for all variables using the scaled Schoenfeld residuals. Clinical covariates included lymph node status (categorical: LN −; LN +), tumor size (categorical: ≤ 20 mm; > 20 mm), and age at diagnosis (categorical: < 50 years old; ≥ 50 years old). The tumor site was included as a stratifying factor in all models. Transcript expression and enrichment pathway scores were evaluated as continuous variables. All continuous variables were centered and scaled (with standard deviation set to one) in the models, such that the HR represents the relative hazard for a one-standard-deviation increase of the variable of interest.

Survival distributions were further evaluated using Kaplan–Meier estimates and the log‐rank test, where transcript expression and enrichment pathway scores were dichotomized using the median value.

## Results

### Cohort characteristics

Clinicopathological features were compared between the primary tumors of PERBC and PESBC (Table 1, and Supplementary Figs. 2-3a). The features that showed significant differences between the groups were tumor size (*p* = 0.000038), tumor grade (*p* = 0.013), the surgical procedure (*p* = 0.0074), type of hormonal treatment (*p* = 0.041), treatment with chemotherapy (*p* = 0.0071), and treatment with radiotherapy (*p* = 0.025; Table 1, and Supplementary Fig. 2). The continuous PR expression did not show significant differences; however, this was seen at the cut-off of 10% (*p* = 0.0039; Table 1 and Supplementary Fig. 3a). Primary tumors from PERBC exhibited higher tumor grades and were more frequently removed by mastectomies. Patients with endocrine-resistant disease more often received adjuvant chemotherapy in the primary setting than sensitive patients. PESBC were, in turn, more often prescribed tamoxifen and at a higher frequency treated with adjuvant radiotherapy. There was no significant difference in TIL scores between the PERBC and PESBC, nor between the relapse and primary tumors of PERBC (Table 1 and Supplementary Table 2).

In the comparison between relapse and primary tumors of PERBC, there were significant differences in PR status (continuous *p* = 0.0048; cut-off of 10% *p* = 0.011), and HER2 status (*p* < 0.00001; Supplementary Table 2, and Supplementary Fig. 3b-c). The patients’ primary tumors showed higher PR expression and were more often HER2-negative/zero than relapse tumors.

### Gene expression analysis

**Fig. 2:**
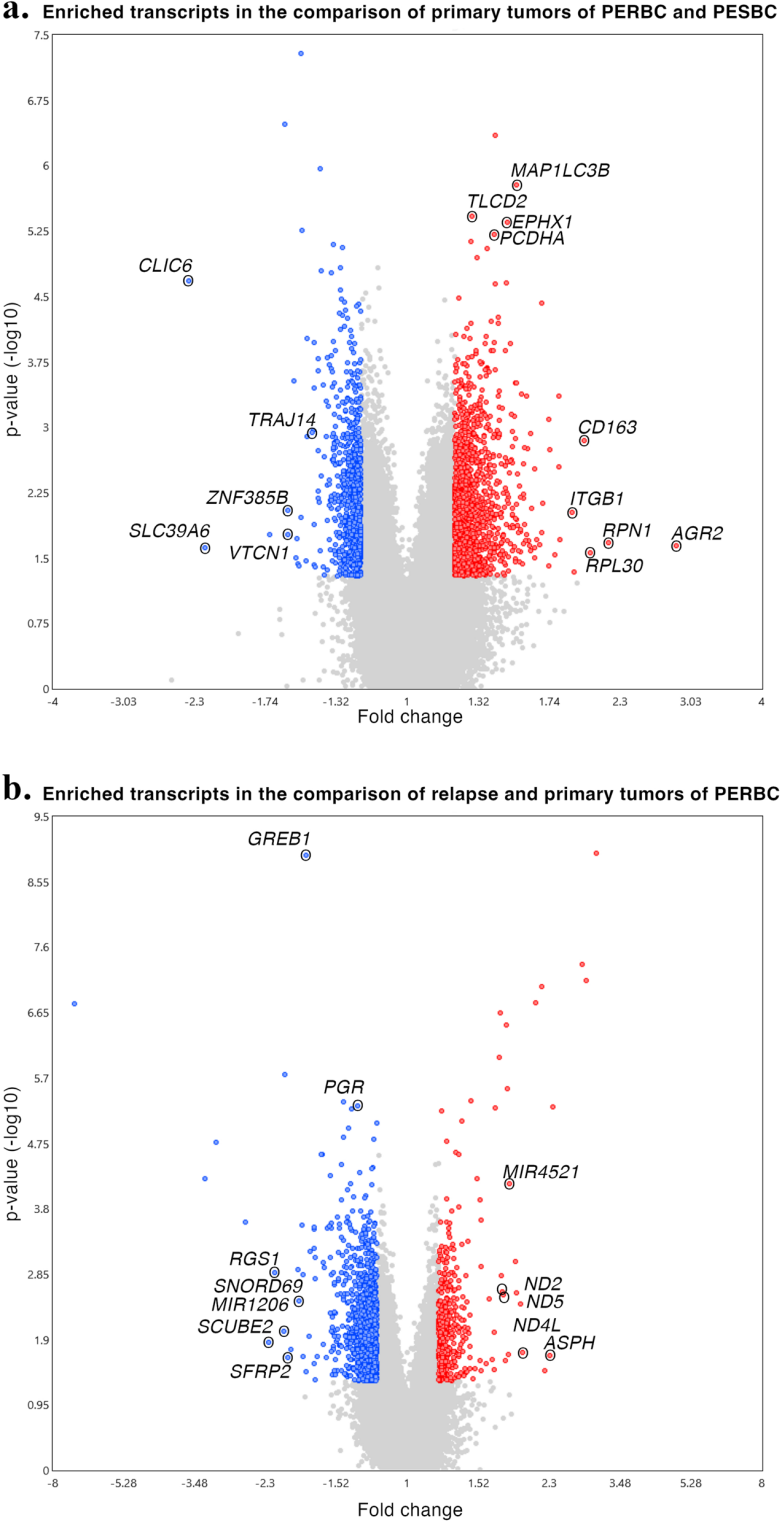
Volcano plots illustrating differential gene expression in the Endoresist cohort. Comparing primary tumors of patients with endocrine-resistant breast cancer (PERBC) versus patients with endocrine-sensitive breast cancer (PESBC) (**a**) and relapse tumors versus primary tumors of PERBC (**b**). The x-axis represents the linear fold change (FC), while the y-axis shows the -log_10_ transformed p-value. Transcripts meeting the thresholds for differential expression (FC >|1.2| and *p* <.05) are highlighted, with upregulated transcripts shown in red and downregulated transcripts in blue. The transcripts with EntrezIDs, meeting the FDR <.05 cut-off in the comparison of relapse and primary tumors of PERBC, and FDR < 0.1 for the comparison of primary tumors of PERBC and PESBC are labeled, together with the top 5 up- and downregulated transcripts of the FC >|1.2| and *p* <.05 threshold of each comparison

To explore potential predisposing transcriptional variations that differentiate the patients’ tumors at diagnosis, prior to endocrine treatment, the expression signatures of primary tumors from PERBC and PESBC were analyzed. The gene *MAP1LC3B* was significantly upregulated in PERBC using FDR-adjusted p-value (< 0.05) and *TLCD2*, *EPHX1*, and *PCDHA* were upregulated with borderline significance (FDR = 0.084-0.092). Applying more lenient cut-offs (*p* < 0.05 and FC >|1.2|), 744 genes (filtered for Entrez IDs) were identified as differentially expressed; 677 upregulated and 67 downregulated in PERBC. This gene list included the downregulation of *PGR* (PR) and upregulation of *MKI67* (Ki67) in PERBC, consistent with the immunohistochemical analyses (Table 1). The PERBC relapse tumors were next compared to their paired primary tumors to investigate potential differential transcriptional changes during ongoing endocrine therapy, aiming to evaluate potential resistance mechanisms. Two genes, the estrogen-regulated *GREB1* and *PGR*, were significantly downregulated (FDR < 0.05) in relapse tumors, corroborating the IHC findings of PR-negative status (Supplementary Table 2). Using the thresholds *p* < 0.05 and FC >|1.2|, 373 genes were differentially expressed in relapse tumors compared to the primary tumors of PERBC, of which 85 were upregulated and 288 downregulated. Volcano plots illustrating the gene expression changes of all transcripts are shown in Fig. 2.

**Fig. 3:**
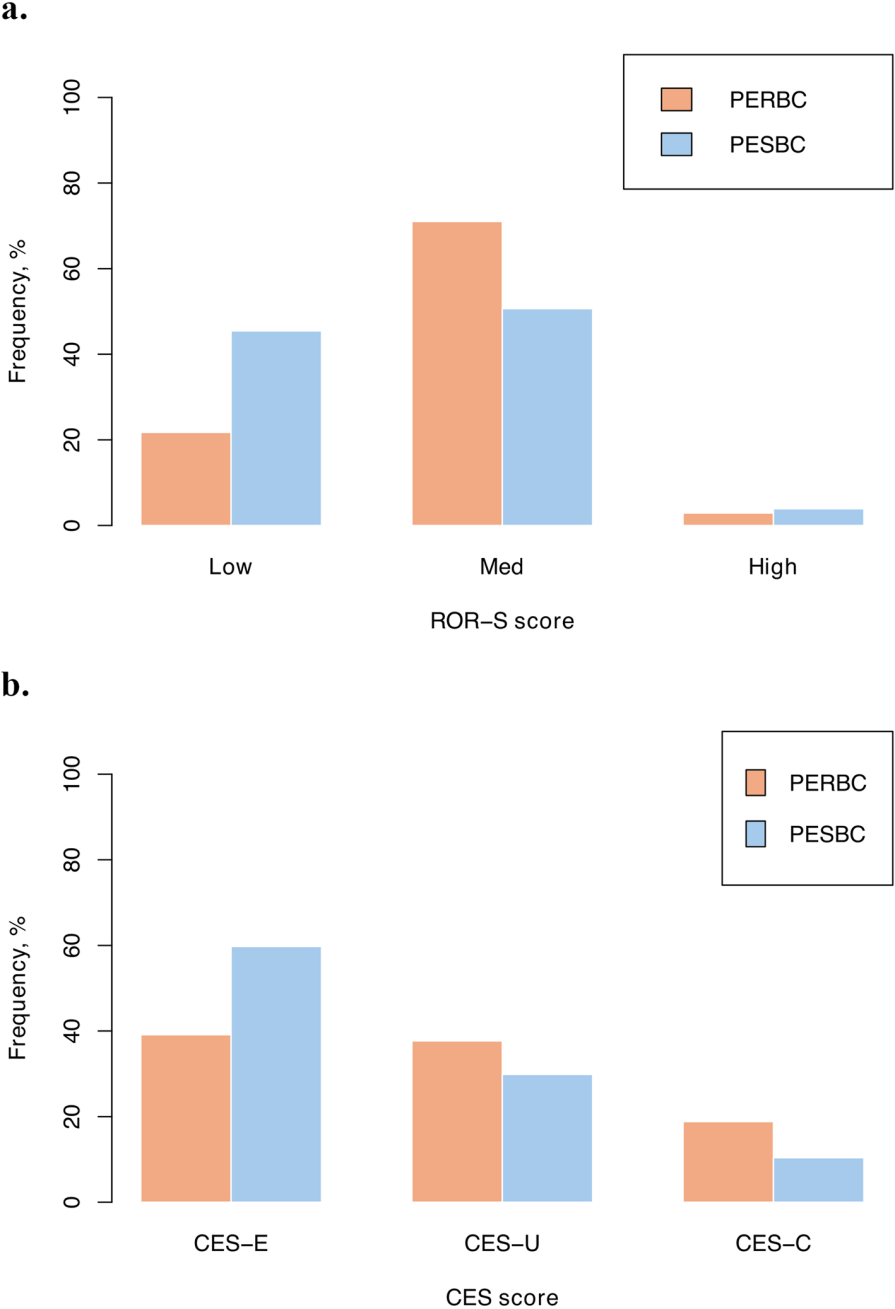
Distribution of risk of recurrence (ROR)-subtype (ROR-S) scores (**a**) and the Chemoendocrine score (CES) (**b**) in the primary tumors of patients with endocrine-resistant breast cancer (PERBC) and patients with endocrine-sensitive breast cancer (PESBC)

**Fig. 4:**
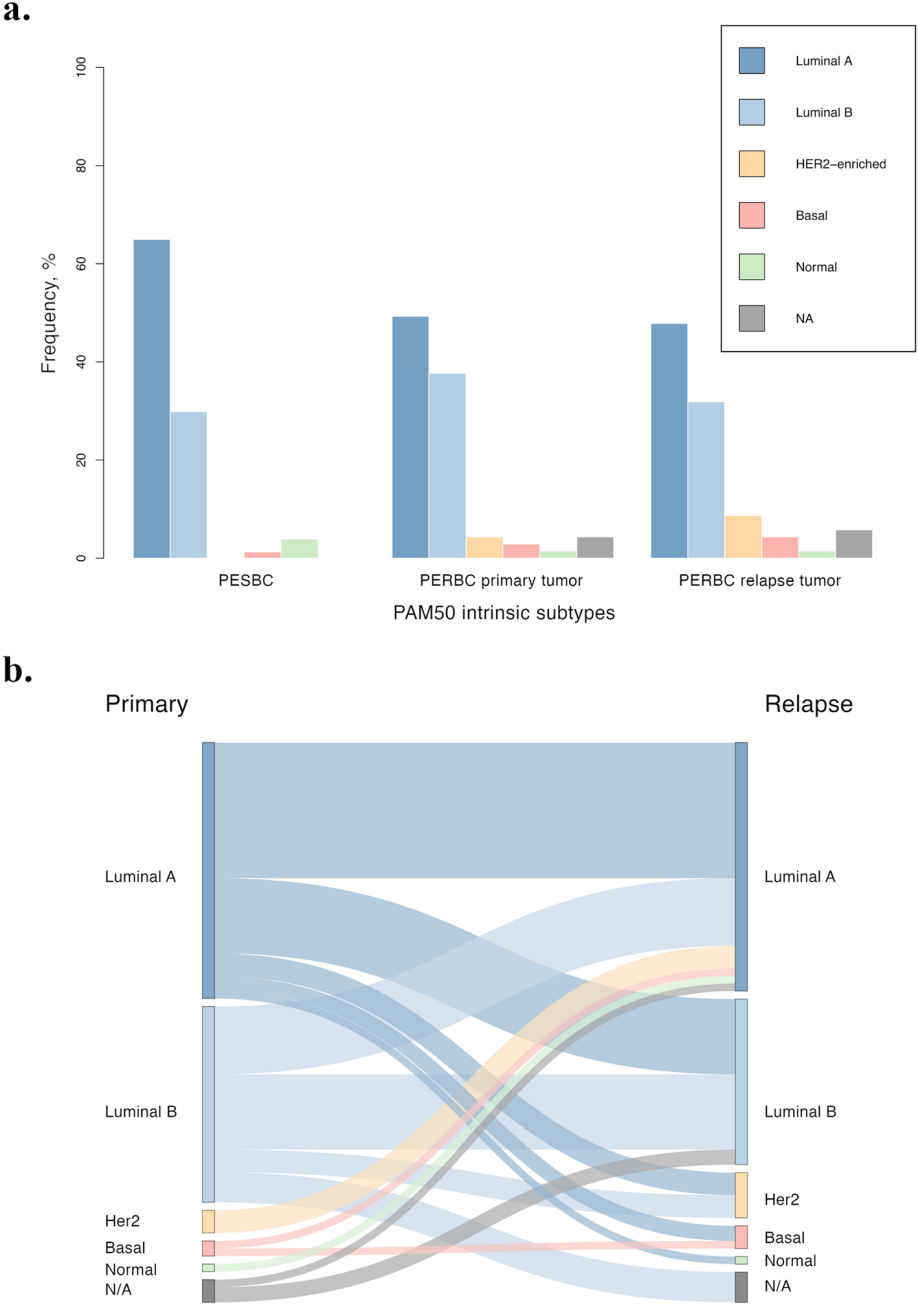
Distribution of PAM50 intrinsic subtypes in the primary tumors of patients with endocrine-sensitive breast cancer (PESBC), patients with endocrine-resistant breast cancer (PERBC), and relapse tumors of PERBC (**a**). Sankey diagram illustrating subtype switching between PERBC primary and relapse tumors (**b**)

The molecular intrinsic subtypes were assessed in the cohort, along with the calculated ROR score and CES, for possible prognostic and predictive information and clinical risk evaluation. The results showed significant differences in the distribution of ROR (*p* = 0.0094), but not for CES (*p* = 0.062) when comparing the primary tumors of PERBC and PESBC (Supplementary Table 3, Fig. 3). There was no significant difference in the PAM50 distribution between the primary tumors of PERBC and PESBC, nor in comparing relapse and primary tumors of PERBC (*p* = 0.14 and *p* = 0.85, respectively; Supplementary Table 3, Fig. 4). Further, 47.83% of the PERBC switched subtype between the primary and relapse tumor (Supplementary Table 3, Fig. 4).

### Gene set enrichment analysis

**Fig. 5:**
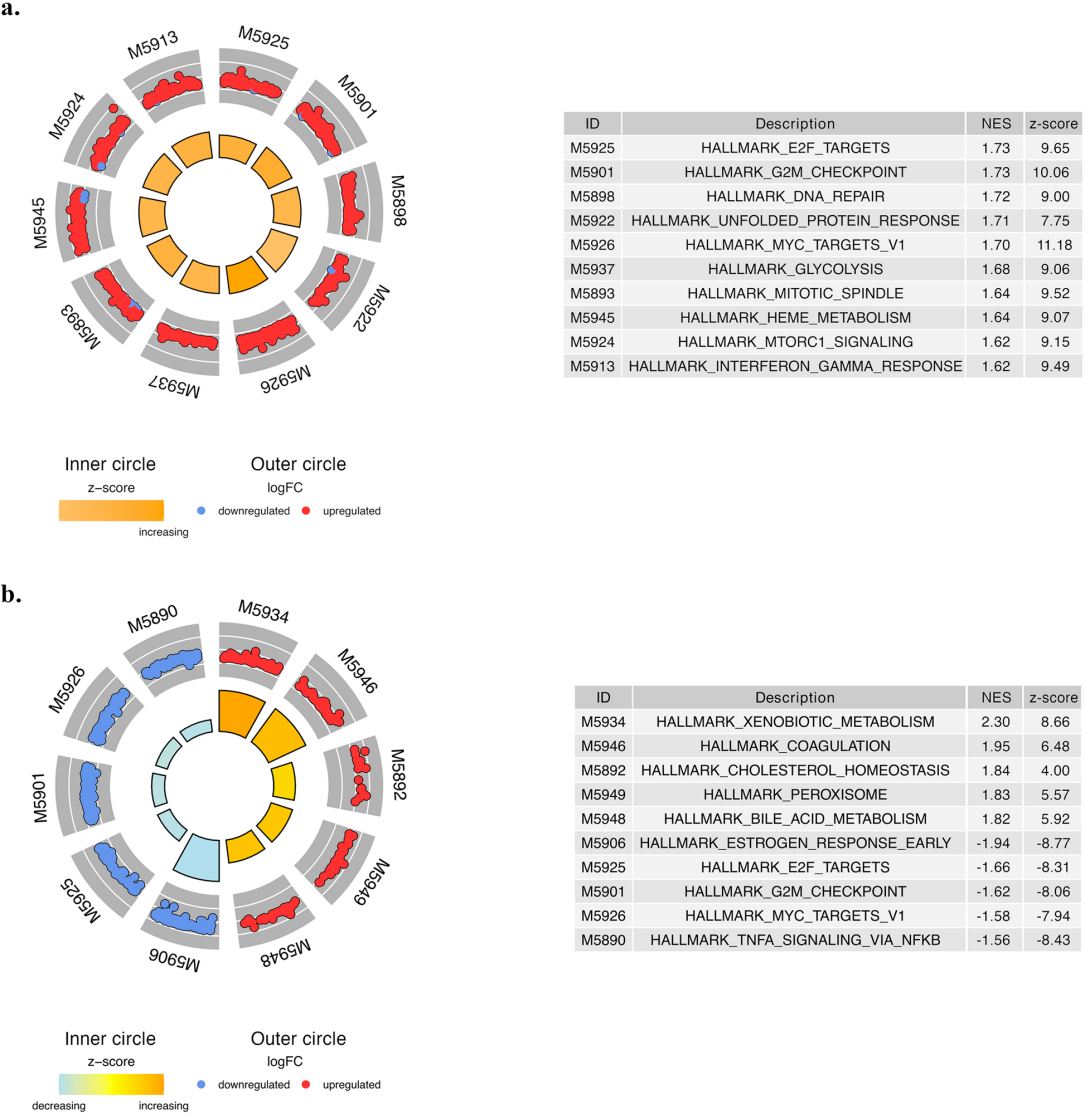
Circle plots of the top differentially regulated Hallmark gene sets from the cohort comparisons, with z-score color visualizations and -log_10_ *p*-value depicted as the height of the inner circle, and fold change of the differentially regulated core enriched genes in each gene set displayed in the outer circle. Upregulated (*N* = 10) gene sets between primary tumors of patients with endocrine-resistant breast cancer (PERBC) and patients with endocrine-sensitive breast cancer (PESBC) (**a**), and upregulated (*N* = 5) and downregulated (*N* = 5) gene sets from the comparison between relapse and primary tumors of PERBC (**b**). LogFC = fold change (log_2_ transformed), NES = normalized enrichment score

**Fig. 6:**
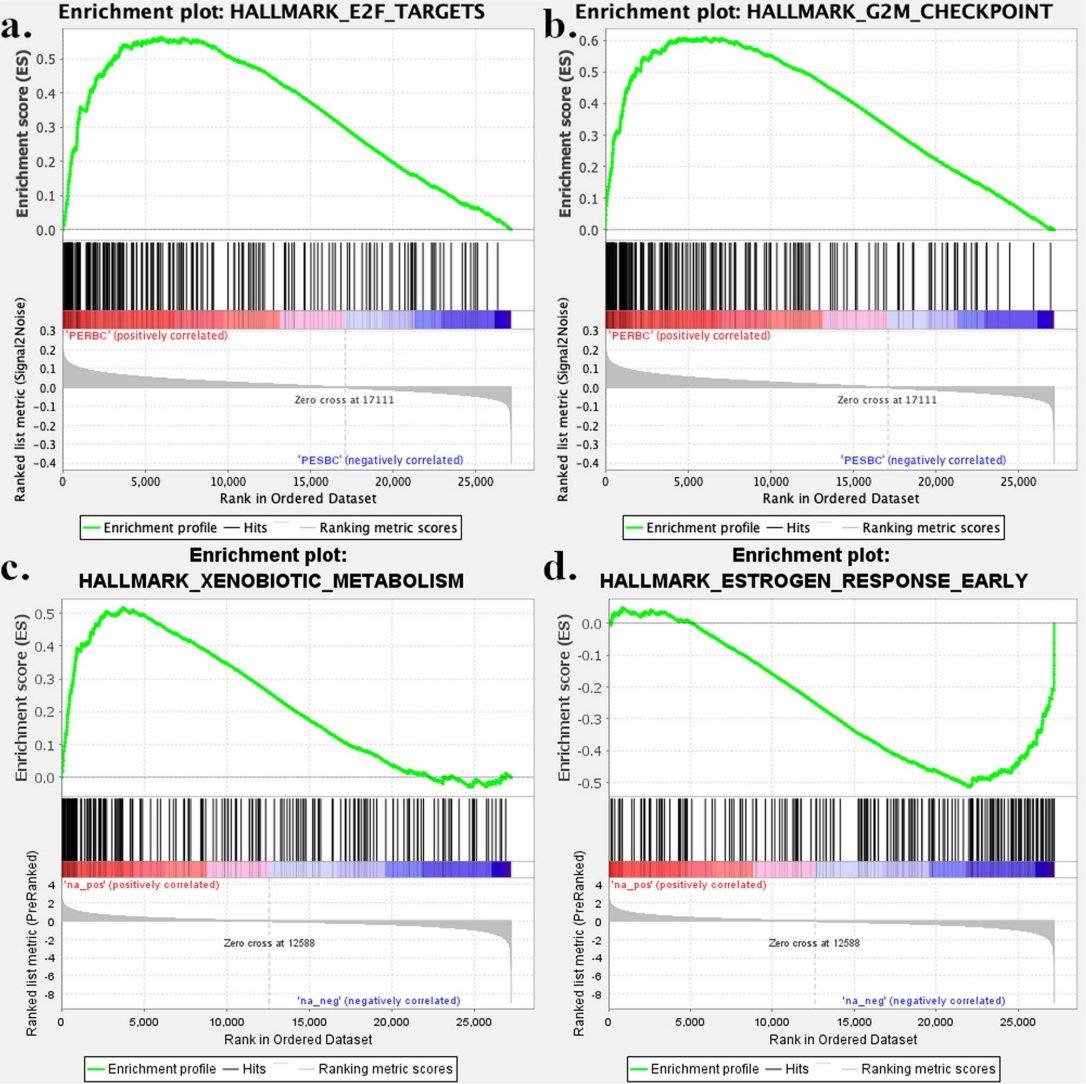
Enrichment plots of the top differentially regulated Hallmark gene sets from the cohort comparisons. Upregulated (*N* = 2) gene sets between primary tumors of patients with endocrine-resistant breast cancer (PERBC) and patients with endocrine-sensitive breast cancer (PESBC); E2F_TARGETS (**a**) and G2M_CHECKPOINT (**b**), and upregulated (*N* = 1) and downregulated (*N* = 1) gene sets from the comparison between relapse and primary tumors of PERBC; XENOBIOTIC_METABOLISM (**c**) and ESTROGEN_RESPONSE_EARLY (**d**)

Gene set enrichment analysis using GSEA was performed with the Hallmark, KEGG, and REACTOME gene sets to explore differentially expressed pathways. The significant gene sets of the Endoresist cohort are presented with NES, nominal *p*-value (NOM p-val), and FDR q-value in the Supplementary Tables 4–7. The top 10 Hallmark gene sets with the highest NES are visualized in Fig. 5. Further, the GSEA enrichment plots of the most enriched gene sets in each comparison are visualized in Fig. 6, and the remaining of the top 10 gene sets in Supplementary Fig. 4 for the comparison of primary tumors of PERBC and PESBC, and in Supplementary Fig. 5 for the comparison of relapse and primary tumors of PERBC. The preranked GSEA analyses for the Hallmark gene sets are demonstrated for the primary ranking metric (signed -log_10_(*p*-value)) in Fig. 5 and Supplementary Table 6a, and described below, and for the secondary metric (log_2_FC) in Supplementary Table 6b.

Comparing primary tumors of PERBC to PESBC from the Endoresist cohort, 49 of the 50 Hallmark gene sets were upregulated in PERBC. Applying a stricter cut-off (FDR q-value of < 0.10), 20 upregulated gene sets were identified in PERBC, with no downregulated gene sets. The top gene sets with the highest NES included E2F targets and G2M checkpoint (Supplementary Table 4). Similarly, analyzing the REACTOME and KEGG gene sets, 78 and 28 were upregulated in PERBC, respectively, and no gene sets were downregulated. Gene sets associated with the cell cycle were most significantly upregulated (Supplementary Tables 5a-b).

In the comparison of relapse tumors to primary tumors of PERBC, Hallmark gene set analysis identified 21 upregulated and 29 downregulated gene sets in relapse tumors. The stricter cut-off yielded 9 upregulated and 7 downregulated gene sets. The top-upregulated gene sets involved xenobiotic metabolism, coagulation, cholesterol homeostasis, and peroxisome. Both early and late estrogen responses (e.g., *GREB1*, *PGR*, *IGFR1*, *MYB*, *NRIP*, *NCOR2*) were downregulated, along with E2F targets (e.g., *H2AX*, *RFC2*, *USP1*), and the G2M checkpoint (e.g., *H2AX*, *TPX2*, *NUP50*, *CDKN1B*) (Fig. 5 and Supplementary Table 6a). Additionally, REACTOME gene set analysis revealed 47 upregulated and 119 downregulated gene sets, and KEGG gene set analysis showed 33 upregulated and no downregulated gene sets. Similarly, drug and lipid metabolism-associated gene sets were differentially upregulated, while epigenetic, ESR (ER)-mediated signaling, and estrogen-dependent gene expression gene sets were downregulated (Supplementary Tables 7a-b).

**Fig. 7:**
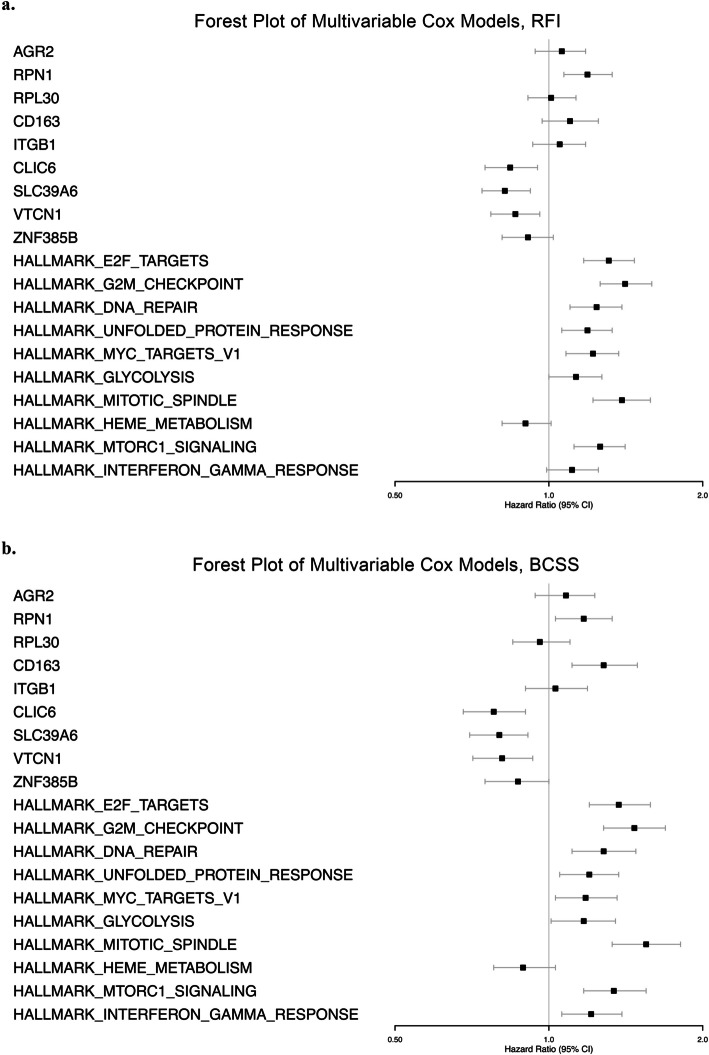
Forest plots of the multivariable Cox regression analyses for Recurrence-Free Interval (RFI; **a**) and breast cancer-specific survival (BCSS; **b**) in the METABRIC cohort, comparing patients with high and low expression of the top differentially expressed genes and gene sets identified from the analysis of primary tumors from patients with endocrine-resistant and endocrine-sensitive breast cancer in the Endoresist cohort

The METABRIC [43] cohort was utilized to investigate the prognostic impact of the differential gene expression profiles. Several genes and Hallmark gene sets that showed significant differential expression between the primary tumors of resistant and sensitive patients in the Endoresist cohort showed significant survival outcomes, based on high versus low expression. From the multivariable Cox regression analyses, four genes and eight gene sets showed significant hazard ratios in terms of RFI depending on a high or low expression of these, and five genes and nine gene sets were significantly associated with BCSS. These findings are visualized in Forest plots in Fig. 7 and in Supplementary Tables 8a-b. Furthermore, the Kaplan–Meier estimates for RFI and BCSS showed similar patterns and are visualized for the genes in Supplementary Figs. 6 and 7, respectively, and for the gene sets in Supplementary Figs. 8 and 9, respectively. Three of the gene sets (E2F targets, G2M checkpoint, and mitotic spindle) involved in the cell cycle appeared with significant differences for HR and CI when comparing both the RFI and BCSS and the Kaplan–Meier estimates; E2F targets (RFI adjusted HR (RFI HRadj) = 1.31, CI = 1.17–1.47, *p* < 0.001), G2M checkpoint (RFI HRadj = 1.41, CI = 1.26–1.59, *p* < 0.001), and mitotic spindle (RFI HRadj = 1.39, CI = 1.22–1.58, *p* < 0.001). Unfolded protein response also showed significant differences (RFI HRadj = 1.19, CI = 1.06–1.33, *p* = 0.003), along with genes *RPN1*, *CLIC6*, *SLC39A6*, and *VTCN1* (RFI HRadj = 1.19, CI = 1.07–1.33, *p* = 0.002, RFI HRadj = 0.84, CI = 0.75–0.95, *p* = 0.004, RFI HRadj = 0.82, CI = 0.74–0.92, *p* < 0.001, and RFI HRadj = 0.86, CI = 0.77–0.96, *p* = 0.009, respectively).

## Discussion

Endocrine resistance in breast cancer is a clinical challenge, with up to one-third of patients not responding to therapy and a majority of these with retained ER expression in their tumors. The issue remains elusive and few clinical markers except ER exist for evaluating which breast cancer patients will benefit from endocrine treatment or are prone to endocrine-resistant disease. Thus, exploring predictive markers and underlying mechanisms could be important to individualize treatment.

In our study, several gene sets and patient and tumor characteristics showed significant differences between the patients already at initial diagnosis, and further through the endocrine-resistant disease progression. The analysis of clinicopathological features of the patients’ primary tumors showed significant distributions of tumor grades, where PERBC had primary tumors with higher grades than PESBC, known to be associated with poor outcome [10, 54]. The resistant patients also had lower PR expression at 10% cut-off and larger tumors, followed by more aggressive treatment regimens. Furthermore, their relapse tumors were more frequently PR-negative than their primary tumors, also associated with a worse outcome [10], and more often of HER2-low status, with debatable predictive and prognostic significance [31].

The PAM50 analysis did not demonstrate significant differences in the distribution of intrinsic subtypes between the patients’ primary tumors (*p* = 0.14), nor for CES (*p* = 0.062) but did so for ROR scores (*p* = 0.0094). However, the patients with resistant disease had a higher proportion of the luminal B subtype, generally associated with a worse prognosis than luminal A, than patients with endocrine-sensitive disease (37.68% and 29.87%, respectively). The luminal A subtype was, in turn, more prevalent in the primary tumors of PESBC than PERBC (64.94% and 49.28%, respectively). This could indicate that patients with luminal B tumors, often indicated for chemotherapy, to a greater extent exhibit endocrine resistance [55]. Patients with luminal A tumors more often recur at a later phase (after 5 years) [7, 56, 57]. Consequently, finding such a large proportion of luminal A tumors in primary tumors with earlier recurrence (PERBC) was unexpected. Previous research has shown a difference in long-term (25 years) distant recurrence-free survival for endocrine-treated patients with luminal A disease at 87% (95% CI, 82%−93%) vs luminal B disease at 67% (95% CI, 56%−82%) [56]. Furthermore, the highest risk for metastasis and greatest benefit of tamoxifen treatment was suggested at 5 years for patients with luminal B disease [56]. Moreover, a large study of over 10,000 breast cancer patients showed a prevalence of locoregional and distant recurrence in 4.8% of patients with luminal A breast cancer and 10.7% among patients with luminal B breast cancer in 10 years of follow-up [57]. The ROR scores are suggested to predict the risk of a late recurrence, and we found a difference already within 5 years [58]. This indicates that the scores could imply earlier recurrence as well. Nonetheless, the intrinsic subtype distribution may not be as favorable in distinguishing patients resistant or responsive to endocrine treatment. The distribution of the intrinsic subtypes was similar between the relapse and primary tumors of PERBC, with no significant difference. Investigating the subtype switching, it is evident that almost half (47.83%) of PERBC switch intrinsic subtypes between the primary and relapse tumors. To our knowledge, no previous research has demonstrated similar results. Though the majority (65.22%) of the recurrences were locoregional (breast or axilla), and the possibility of a new cancer and a new subtype, instead of a subtype switch, cannot be excluded. The CES showed a higher frequency of the CES-E group in patients with endocrine-sensitive disease (59.74%) than those with endocrine-resistant disease (39.13%), although not significant. This suggests a greater chance of responding to endocrine therapy, consistent with the outcome of these patients [50, 51].

Endocrine treatment has previously foremost focused on the ER and not on downstream signaling or other regulator units [59]. ER signaling primarily operates through genomic pathways, wherein it binds to estrogen-responsive elements in the genome and regulates gene transcription upon ligand activation [60, 61]. ER also engages in non-genomic functions outside the nucleus, interacting with growth factor receptors and signaling molecules to activate downstream cascades, including the PI3K/AKT/mTOR and MAPK pathways [22, 27]. Activation of these pathways has been linked to reduced ER levels and activity, poor prognosis, and resistance to endocrine therapy in experimental models [62, 63]. Precision medicine offers new potentials in cancer diagnostics and treatments including therapies such as CDK4/6 inhibitors, utilized as adjuvant therapy for ER + patients, with reductions of risk of recurrence, and PI3K and mTOR inhibitors for advanced metastatic ER + patients, showing promising results, especially in terms of progression-free survival [3, 64–66]. Our study finds that gene sets involved in the cell cycle and PI3K/AKT/mTOR signaling differentiate resistant and sensitive tumors at diagnosis. These pathways may thus be possible predictive and prognostic markers, as well as targets for additional therapy, such as these inhibitors, perhaps even early in the disease progression.

When comparing the primary tumors of patients with resistant and sensitive BC, PERBC showed upregulation in several gene sets involved in the cell cycle and proliferation, such as E2F targets, G2M checkpoint, MYC targets, and the mitotic spindle compared to PESBC. Altering the cell cycle and driving proliferation are known Hallmarks of Cancer [67] and suggested mechanisms for therapy resistance [68, 69]. Interestingly, these were upregulated already at diagnosis and might have a predictive response potential. Further, evaluating the external cohort also demonstrated a significant difference when comparing HR, RFI, and BCSS for patients with high or low expression of Hallmark gene sets E2F targets, G2M checkpoint, and mitotic spindle, favoring the patients with low expression. Similarly, previous research on patients with tumors that progressed or remained stable after four months of endocrine treatment also identified upregulations of proliferation and cell cycle pathways in non-responders [70]. Another study has shown that proliferation and cell-cycle pathways are inhibited in patients with both responding and non-responding tumors, however to a greater extent in the responders [71]. The E2F targets and G2M checkpoint Hallmark gene sets have been associated with poor prognosis when assessing gene expression of The Cancer Genome Atlas (TCGA) and METABRIC data [72, 73]. Viewing the results from the relapse tumors compared to primary tumors, the cell cycle gene sets are instead downregulated. This may be due to treatment effects or the evident upregulation in the primary tumors but may also reflect shifts toward other pathways, more important for the tumor cells’ survival when under ongoing endocrine therapy. No patients in the cohort were treated with CDK4/6 inhibitors. Importantly, no predictive marker for CDK4/6 inhibitor exists today. Moreover, resistance to the inhibitors is evident and new strategies are needed in order to distinguish the patients suited for the therapy. Perhaps these gene sets can be further investigated examining CDK4/6 inhibitor resistance, in order to evaluate their predictive and prognostic potential.

The gene *RPN1* was significantly upregulated in the primary tumors of PERBC compared to PESBC in the Endoresist cohort and a low expression of this gene was also significantly associated with a better outcome in the external METABRIC cohort, and has previously been suggested as a potential diagnostic marker and target for treatment [74]. Expression of *SLC39A6*, which, in turn, was seen as downregulated in the Endoresist cohort and with a high expression associated with a better outcome in the external cohort, has been shown to indicate a better prognosis for patients with luminal breast cancers [75].

In the comparison between relapse and primary tumors of PERBC, an evident downregulation could be seen in both gene sets of early and late estrogen response, as well as ESR-dependent signaling and estrogen-dependent gene expression. Previous studies have shown that low scores of the estrogen response gene sets correlate to a worse prognosis and outcome in both primary and metastatic breast cancer [76, 77]. Nevertheless, it cannot be ruled out that the effect on the pathways is at least partially a result of ongoing therapy. Furthermore, this therapy effect may pave the way for upregulating other signaling pathways to enhance tumor progression and growth. Upregulations, and thus possible tumor drivers, were in turn seen in metabolism-associated gene sets such as xenobiotic metabolism and drug metabolism by cytochrome P450, which could indicate a greater metabolism of medications such as endocrine therapy in the relapse tumors, driving resistance. The cytochrome family enzymes are involved in both xenobiotic metabolism for pharmaceuticals and endogenous elements such as cholesterol and estrogen and could consequently contribute to drug resistance [78, 79]. Other drivers of resistance suggested in our data are fatty acid, cholesterol and steroid hormone synthesis and metabolism, all of which have shown associations to endocrine resistance [23, 26, 28, 80]. The gene sets were not significantly altered in the primary setting, and thus their predictive quality is questionable but could be assessed for resistance development. Moreover, additional investigations are needed in order to decipher potential intrinsic or acquired resistance of these gene sets.

Comparing the two ranking approaches of the GSEA preranked analysis of relapse and primary tumors of PERBC, the results conveyed an overall preservation of the key gene sets. This indicates a robustness of our biological conclusions. We thus chose to primarily demonstrate the initial analysis of signed –log₁₀(p‑value), which has been well studied [81, 82]. However, oxidative phosphorylation emerged as the top upregulated gene sets in patients relapse tumors in the log₂FC approach, as seen in Supplementary Table 6b. This further emphasizes metabolism as a tumor driver in our results. Previous research has identified oxidative phosphorylation as a key pathway of metastatic ER + breast cancer by evaluations of patients’ tumors, metastasis-derived patient derived xenografts and isogenic cell lines [83]. Further, the pathway has been shown to implicate CDK4/6 inhibitor resistance and treatment with an oxidative phosphorylation inhibitor has shown tumor growth inhibition in endocrine and palbociclib resistant patient derived xenografts [83].

This study involved stringent inclusion criteria to create a specific cohort with intricate characteristics and to minimize interfering variables, resulting in a small but consistent cohort. A study with a larger population is thus needed to establish and verify the results and to examine potential subsets of endocrine-resistant patients and their characteristics. Patient compliance with therapy is a known problem in healthcare, and was controlled through patients’ medical journals by medical professionals in the research team. Further, confounding factors such as the sampling of PERBC and PESBC are already known to carry different properties and subsequent patient outcomes.

Although Affymetrix Microarray has shown to be a robust method for this type of archived material [84], errors due to data quality may be present, especially in terms of degraded RNA, may be present. Moreover, bulk RNA was analyzed, and future studies on for example single-cell analysis or spatial transcriptomics could aid to ascertain the findings.

When analyzing the data, few genes showed a differential expression at the initial cut-off of FC >|2|, a stringent, conventional microarray threshold. A more lenient threshold was chosen to capture subtler, potentially meaningful shifts, especially important given the RNA quality and smaller cohort size. FC >|1.2| was established based on the arms appearance of the volcano plot, warranting further exploration of the genes in the 1.2–2 FC range. The more lenient threshold, however, increases the risk of false‑positive results. This was mitigated by requiring *p* < 0.05, and further by the downstream GSEA analysis, investigating coordinated pathway signals rather than relying on single genes.

In order to diminish possible batch effects, the patient selection and inclusion were handled and rechecked by two individuals. Several measures were taken to ensure that the merging of the cohorts would give reliable results. The batch effects were tested in several QC steps in the TAC software, and all analyses in TAC and downstream GSEA was compared in each step to diminish inaccuracies in terms of under- and/or overcorrection, and to ensure consistent findings. To our knowledge, no similar study has investigated the transcriptional patterns of patients with verified endocrine-resistant relapse tumors during ongoing treatment in paired comparison to their primary tumors. Though, the differences in the treatments between the primary tumors of PERBC and PESBC may affect the results, and a larger population would be needed for testing these comparisons. Mechanistic studies comparing tamoxifen and AI-treated breast cancer cells could also help elucidate possible differences. The issue of potential intrinsic and acquired endocrine resistance also needs assessment, where tumor sampling at different time points may help guide investigations on whether resistance can be assessed already at primary diagnosis or if treatment selection and pressure may induce resistance mechanisms.

The PESBC were selected as potential responders based on no progression after 10 years of follow-up. However, endocrine resistant disease may arise after several more years [56], and an extended follow-up may be needed to compare true endocrine resistance patterns. The PERBC and PESBC were known to exhibit differences prior to gene expression analysis, and this may cause potential biases in the results. The differential expression analysis compares resistant versus sensitive ER⁺ tumors of patients, rather than tumor versus normal breast tissue. Consequently, the differential gene expression reflects relative changes between two tumor states and may not capture absolute tumor‑specific expression shifts. Future studies incorporating matched normal controls will be necessary to fully contextualize these findings.

Members of the upregulated gene sets in the relapse setting of PERBC may further be queried against existing databases to prioritize existing small-molecule inhibitors that could be validated in mechanistic studies. Ex vivo organoid models, derived from patient tumors, are emerging as promising tools for modeling endocrine resistance. These models can capture aspects of the tumor architecture and microenvironment and may allow for functional testing of drug responses in a patient-specific context [85]. Future research may leverage both preclinical functional systems, such as patient derived xenografts and organoids, and prospective clinical cohorts to validate and dissect the pathways identified in our transcriptomic data. These efforts may help move from correlative observations to mechanistic understanding and ultimately toward individualized treatment strategies.

Our transcriptome data is consistent with the immunological assays (e.g., PR) and, along with clinicopathological assessments, adheres to previous studies, supporting its reliability. Our data suggests specific pathways involved in tumor progression and resistant traits that may pave the way for further studies of endocrine resistance development and predictive markers.

## Conclusions

In conclusion, this study demonstrates that distinct clinicopathological and transcriptional profiles are evident in tumors of patients with verified endocrine-resistant breast cancer. In the analysis, tumor grade and intrinsic subtype risk scores differentiated patients’ tumors already at diagnosis. Moreover, metabolic pathways emerge as possible resistance mechanisms, together with altered cell-cycle pathways. These results may help the continuous investigation of potential prognostic and predictive markers for endocrine-resistant breast cancer, and shift towards alternative treatments.

## Supplementary Information

Supplementary Material 1.

## Data Availability

The microarray data analyzed in the current study is uploaded to and can be requested from the Swedish National Data Service (SND) with DOI: 10.48723/kd85-8s18.

## Abbreviations

AI: Aromatase Inhibitors
AKT: Protein kinase B
BCSS: Breast Cancer-Specific Survival
BRISQ: Biospecimen reporting for improved study quality
CC: Correlation coefficient
CDK4/6: Cyclin-dependent kinase 4/6
CES: Chemoendocrine Score
CES-C: Chemoendocrine Score chemo-sensitive
CES-E: Chemoendocrine Score endocrine-sensitive
CES-U: Chemoendocrine Score uncertain
CI: Confidence interval
ER: Estrogen receptor
FC: Fold change
FDR: False discovery rate
FFPE: Formalin-fixed paraffin-embedded
GNRHA: Gonadotropin-releasing hormone agonists
GSEA: Gene Set Enrichment Analysis
GSVA: Gene Set Variation Analysis
HER2: Human epidermal growth factor receptor 2
HR: Hazard ratio
IHC: Immunohistochemistry
ISH: In situ hybridization
KEGG: Kyoto Encyclopedia of Genes and Genomes
LN: Lymph node
NES: Normalized Enrichment Score
NHG: Nottingham Histologic Grade
PAM50: Prediction Analysis of Microarray 50
PERBC: Patients with endocrine-resistant breast cancer
PESBC: Patients with endocrine-sensitive breast cancer
PH: Proportional hazards
PI3K: Phosphoinositide 3-kinase
PR: Progesterone receptor
QC: Quality control
REMARK: Reporting Recommendations for Tumor Marker Prognostic Studies
RFI: Recurrence-Free Interval
RFI HRadj: RFI adjusted HR
RIN: RNA integrity number
RMA: Robust Multi-chip Analysis
ROR: Risk of recurrence
ROR-S: Risk of recurrence-subtype
SST: Signal Space Transformation
TAC: Transcriptome Analysis Console
TAM: Tamoxifen
TCGA: The Cancer Genome Atlas
TILs: Tumor-infiltrating lymphocytes
