# Supplementary material for: Transcriptomic profiles of endocrine-resistant breast cancer
Source: BMC Cancer. 2025 Oct 13;25:1556. doi: 10.1186/s12885-025-14826-1 (PMC12516873; doi:10.1186/s12885-025-14826-1)
Supplement: Supplementary file 1 — Supplementary Material 1. [file 12885_2025_14826_MOESM1_ESM.pdf]

Supplementary Information

Transcriptomic profiles of endocrine-resistant breast cancer

Caroline Schagerholm Stanev, Emmanouil G. Sifakis, Linnea Hases, Xinsong Chen, Cecilia Williams, Stephanie Robertson, Johan Hartman

Table of contents

Supplementary Figures:

Supplementary Figure 1. Consort diagram showing the selection of the Endoresist cohort.
Supplementary Figure 2. Distribution of tumor (pT) stage (a), histologic grade (b), surgical procedure (c), type of adjuvant hormonal therapy (d), treatment with chemotherapy (e), and treatment with radiotherapy (f) in primary tumors of patients with endocrine-resistant breast cancer (PERBC) and patients with endocrine-sensitive breast cancer (PESBC).
Supplementary Figure 3. Distribution of PR status with a cut-off of 10% (a) in primary tumors of patients with endocrine-resistant breast cancer (PERBC) and patients with endocrine-sensitive breast cancer (PESBC), and distribution of PR status with a cut-off of 10% (b) and HER2 status (b) in relapse and primary tumors of patients with endocrine-resistant breast cancer (PERBC).
Supplementary Figure 4. Enrichment plots of the remaining top differentially regulated Hallmarks gene sets from the comparison between primary tumors of patients with endocrine-resistant breast cancer and patients with endocrine-sensitive breast cancer.
Supplementary Figure 5. Enrichment plots of the remaining top differentially regulated Hallmarks gene sets from the comparison between relapse and primary tumors of patients with endocrine-resistant breast cancer.
Supplementary Figure 6. Kaplan-Meier plots of the of the recurrent-free interval (RFI) of the METABRIC cohort comparing high (red) and low (blue) expression of the top 5 up-and downregulated genes (note, except *TRAJ14*, Entrez ID: 28741, not present in the METABRIC analysis) in the Endoresist cohorts' comparison of primary tumors of endocrine-resistant and endocrine-sensitive patients.
Supplementary Figure 7. Kaplan-Meier plots of the breast cancer-specific survival (BCSS) of the METABRIC cohort comparing high (red) and low (blue) expression of the top 5 up- and downregulated genes (note, except *TRAJ14*, Entrez ID: 28741, not present in the METABRIC analysis) in the Endoresist cohorts' comparison of primary tumors of endocrine-resistant and endocrine-sensitive patients.
Supplementary Figure 8. Kaplan-Meier plots of the recurrent-free interval (RFI) of the METABRIC cohort comparing high (red) and low (blue) expression of the top 10 upregulated gene sets in the Endoresist cohorts' comparison of primary tumors of endocrine-resistant and endocrine-sensitive patients.
Supplementary Figure 9. Kaplan-Meier plots of the breast cancer-specific survival (BCSS) of the METABRIC cohort comparing high (red) and low (blue) expression of the top 10 upregulated gene sets in the Endoresist cohorts' comparison of primary tumors of endocrine-resistant and endocrine-sensitive patients.

Supplementary Tables:

Supplementary Table 1. Biospecimen Reporting for Improved Study Quality (BRISQ) report of the study.
Supplementary Table 2. Clinicopathological characteristics of all primary and relapse tumors from patients with endocrine-resistant breast cancer (PERBC).
Supplementary Table 3. Signature information of all patients in the Endoresist cohort, divided into tumor categories.
Supplementary Table 4. Gene set enrichment Analysis (GSEA) output of the Hallmark gene set comparisons, from the analysis of the merged cohorts' primary tumors of endocrine-resistant patients compared to endocrine-sensitive patients.
Supplementary Table 5a. Gene set enrichment Analysis (GSEA) output of the REACTOME gene set comparisons, from the analysis of the merged cohorts' primary tumors of endocrine-resistant patients compared to endocrine-sensitive patients.
Supplementary Table 5b. Gene set enrichment Analysis (GSEA) output of the KEGG gene set comparisons, from the analysis of the merged cohorts' primary tumors of endocrine-resistant patients compared to endocrine-sensitive patients.
Supplementary Table 6a. Gene set enrichment Analysis (GSEA) output of the Hallmark gene set comparisons, from the analysis of the merged cohorts' endocrine-resistant patients relapse and primary tumors, using the preranked GSEA with the primary ranking metric ($-\log_{10}$(p-value)).
Supplementary Table 6b. Gene set enrichment Analysis (GSEA) output of the Hallmark gene set comparisons, from the analysis of the merged cohorts' endocrine-resistant patients relapse and primary tumors, using the preranked GSEA with the secondary ranking metric ($\log_2$FC).
Supplementary Table 7a. Gene set enrichment Analysis (GSEA) output of the REACTOME gene set comparisons, from the analysis of the merged cohorts' endocrine-resistant patients relapse and primary tumors.
Supplementary Table 7b. Gene set enrichment Analysis (GSEA) output of the KEGG gene set comparisons, from the analysis of the merged cohorts' endocrine-resistant patients relapse and primary tumors.
Supplementary Tables 8a-b.
Multivariable Cox regression results and parameters for recurrent-free interval (RFI) (a) and breast cancer-specific survival (BCSS) (b) of the METABRIC cohort analysis comparing the patients with high and low expression of the top 5 up- and downregulated genes (except *TRAJ14*, Entrez ID: 28741, not present in the METABRIC analysis) and top 10 differentially expressed Hallmark gene sets, obtained from the analysis of primary tumors of endocrine-resistant and endocrine-sensitive patients in the Endoresist cohort.

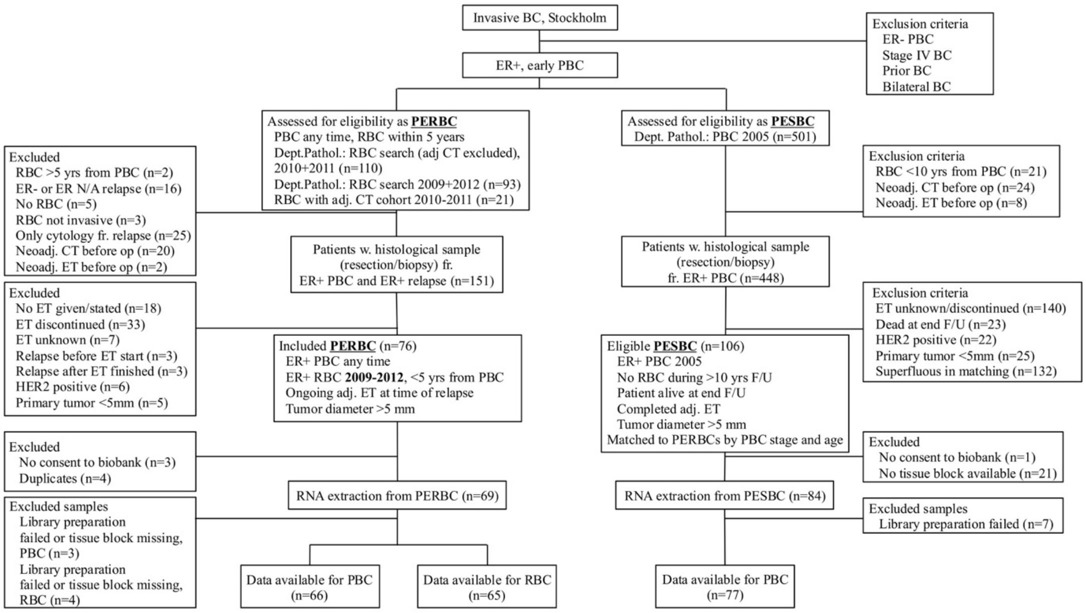

Supplementary Figure 1. Consort diagram showing the selection of the Endoresist cohort. In the first cohort, assessing patients diagnosed in 2010-2011, patients treated with adjuvant chemotherapy in the primary setting were initially excluded to diminish treatment effects. The effect of chemotherapy given several years before the recurrent tumor was re-evaluated to be minor, and these patients were thus included in the second cohort. The second cohort included patients diagnosed in 2009 and 2012, treated both with and without adjuvant chemotherapy. In the first cohort, the ratio between patients with endocrine-sensitive breast cancer (PESBC) and patients with endocrine-resistant breast cancer (PERBC) was included in a 2:1 manner, and in the second cohort 1:1 to obtain a sufficient number of patients. Due to many more patients being eligible as PESBC than PERBC, not all eligible patients were included in the matching. These were randomly selected for their respective criteria and the remainder thus excluded as “superfluous in matching”. BC=breast cancer, PBC=primary breast cancer, ER=estrogen receptor, PERBC=patients with endocrine-resistant breast cancer, RBC, recurrent breast cancer, Dept.=department, Pathol.=Pathology, adj.=adjuvant, CT=chemotherapy, PESBC=patients with endocrine-sensitive breast cancer, N/A=data not available, fr.=from, neoadj.=neoadjuvant, ET=endocrine therapy, op.=operation, surgery, HER2=human epidermal growth factor receptor 2, F/U=follow-up.

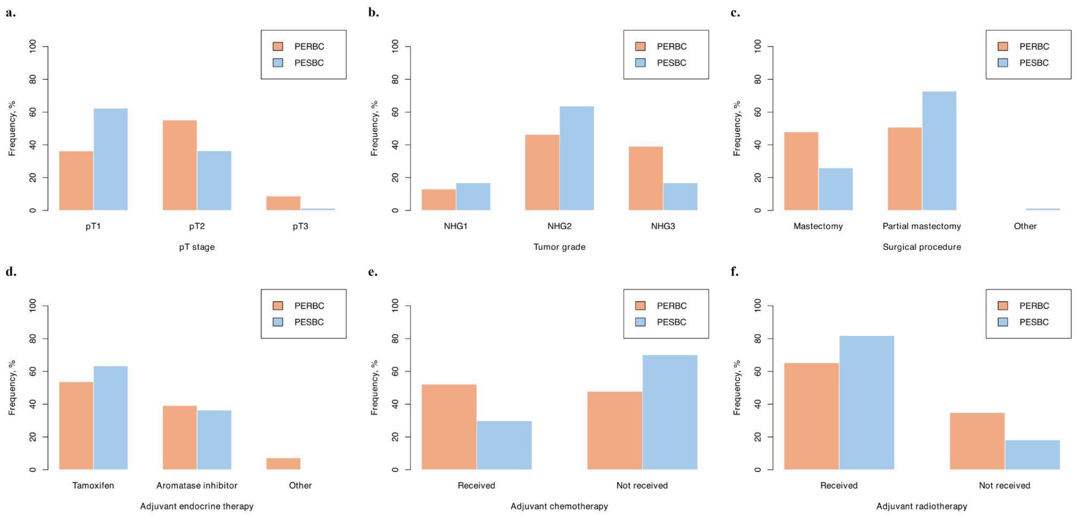

Supplementary Figure 2. Distribution of tumor (pT) stage (a), histologic grade (b), surgical procedure (c), type of adjuvant hormonal therapy (d), treatment with chemotherapy (e), and treatment with radiotherapy (f) in primary tumors of patients with endocrine-resistant breast cancer (PERBC) and patients with endocrine-sensitive breast cancer (PESBC).

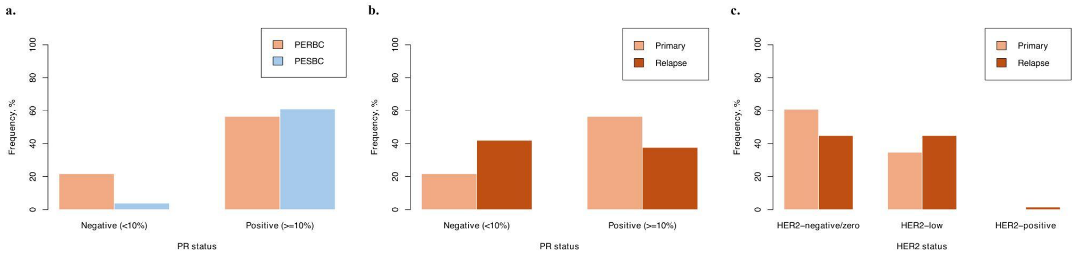

Supplementary Figure 3. Distribution of PR status with a cut-off of 10% (a) in primary tumors of patients with endocrine-resistant breast cancer (PERBC) and patients with endocrine-sensitive breast cancer (PESBC), and distribution of PR status with a cut-off of 10% (b) and HER2 status (b) in relapse and primary tumors of patients with endocrine-resistant breast cancer (PERBC).

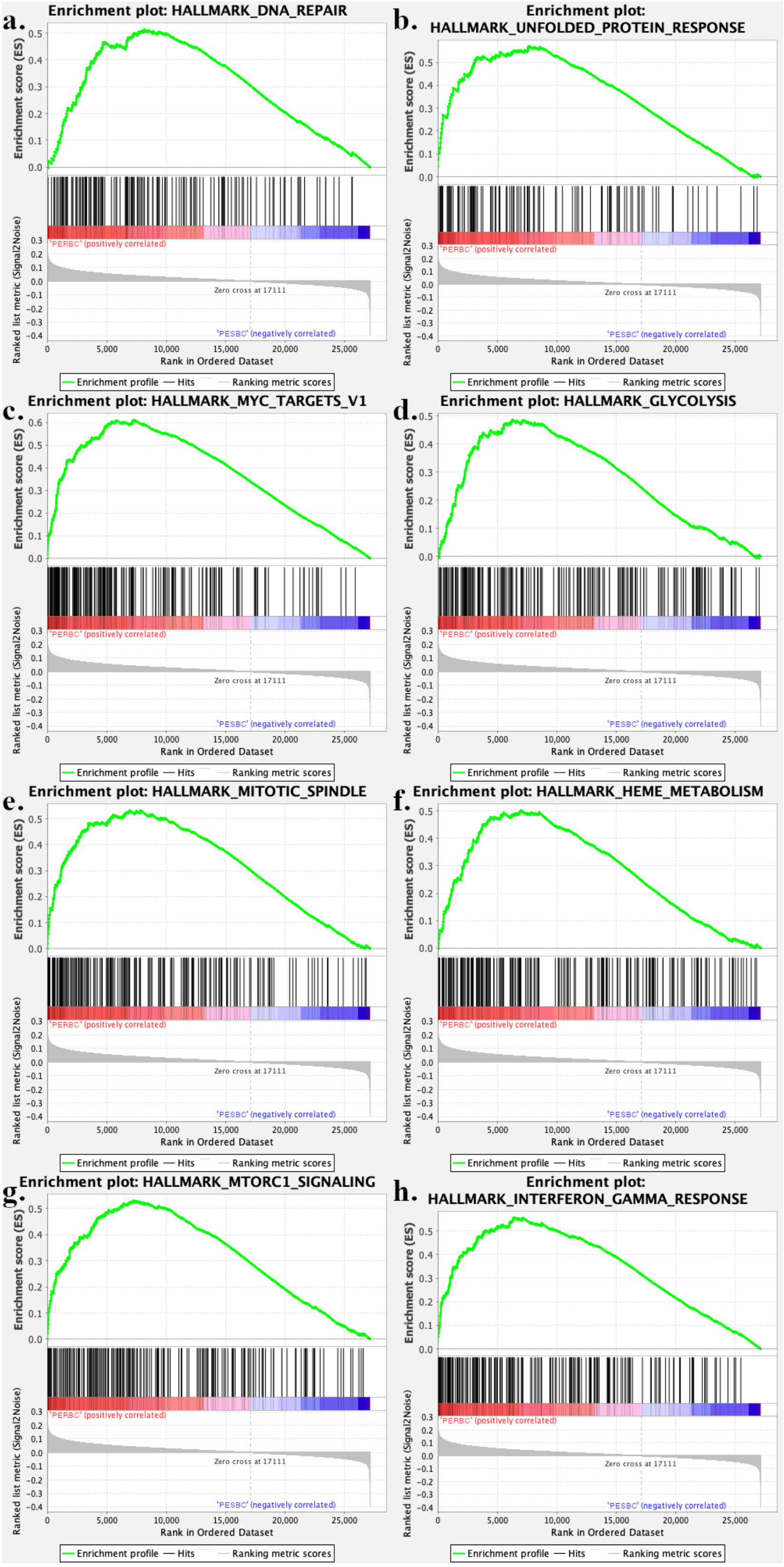

Supplementary Figure 4. Enrichment plots of the remaining top differentially regulated Hallmarks gene sets from the comparison between primary tumors of patients with endocrine-resistant breast cancer (PERBC) and patients with endocrine-sensitive breast cancer (PESBC). Upregulated (8) gene sets; DNA_REPAIR (a), UNFOLDED_PROTEIN_RESPONSE (b), MYC_TARGETS_V1 (c), GLYCOLYSIS (d), MITOTIC_SPINDLE (e), HEME_METABOLISM (f), MTORC1_SIGNALING (g), and INTERFERON_GAMMA_RESPONSE (h).

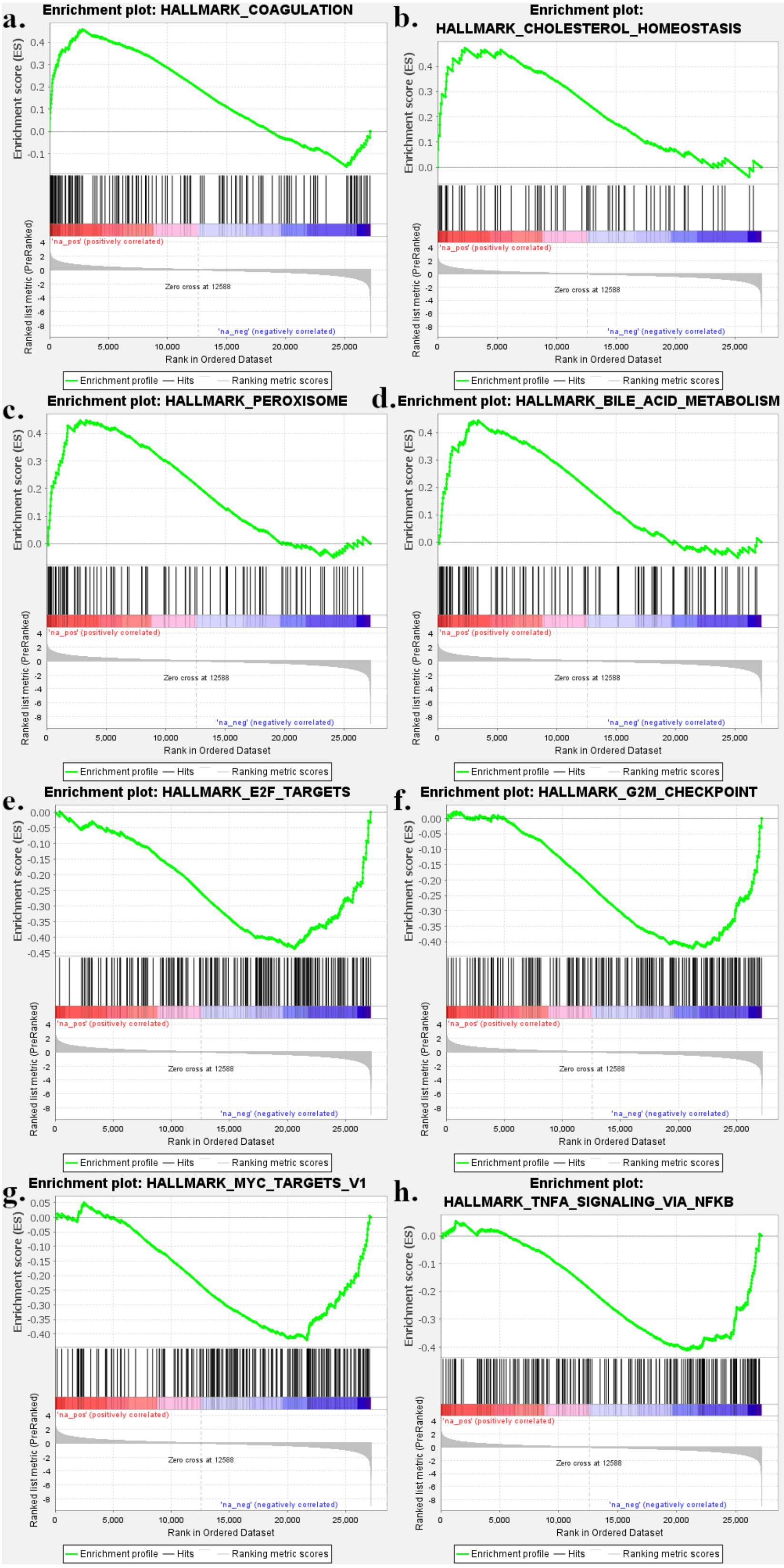

Supplementary Figure 5. Enrichment plots of the remaining top differentially regulated Hallmarks gene sets from the comparison between relapse and primary tumors of patients with endocrine-resistant breast cancer (PERBC). Upregulated (N=4) gene sets; COAGULATION (a), CHOLESTEROL_HOMEOSTASIS (b), PEROXISOME (c), and BILE_ACID_METABOLISM (d), and downregulated (N=4) gene sets; E2F_TARGETS (e), G2M_CHECKPOINT (f), MYC_TARGETS_V1 (g), and TNFA_SIGNALING_VIA_NFKB (h).

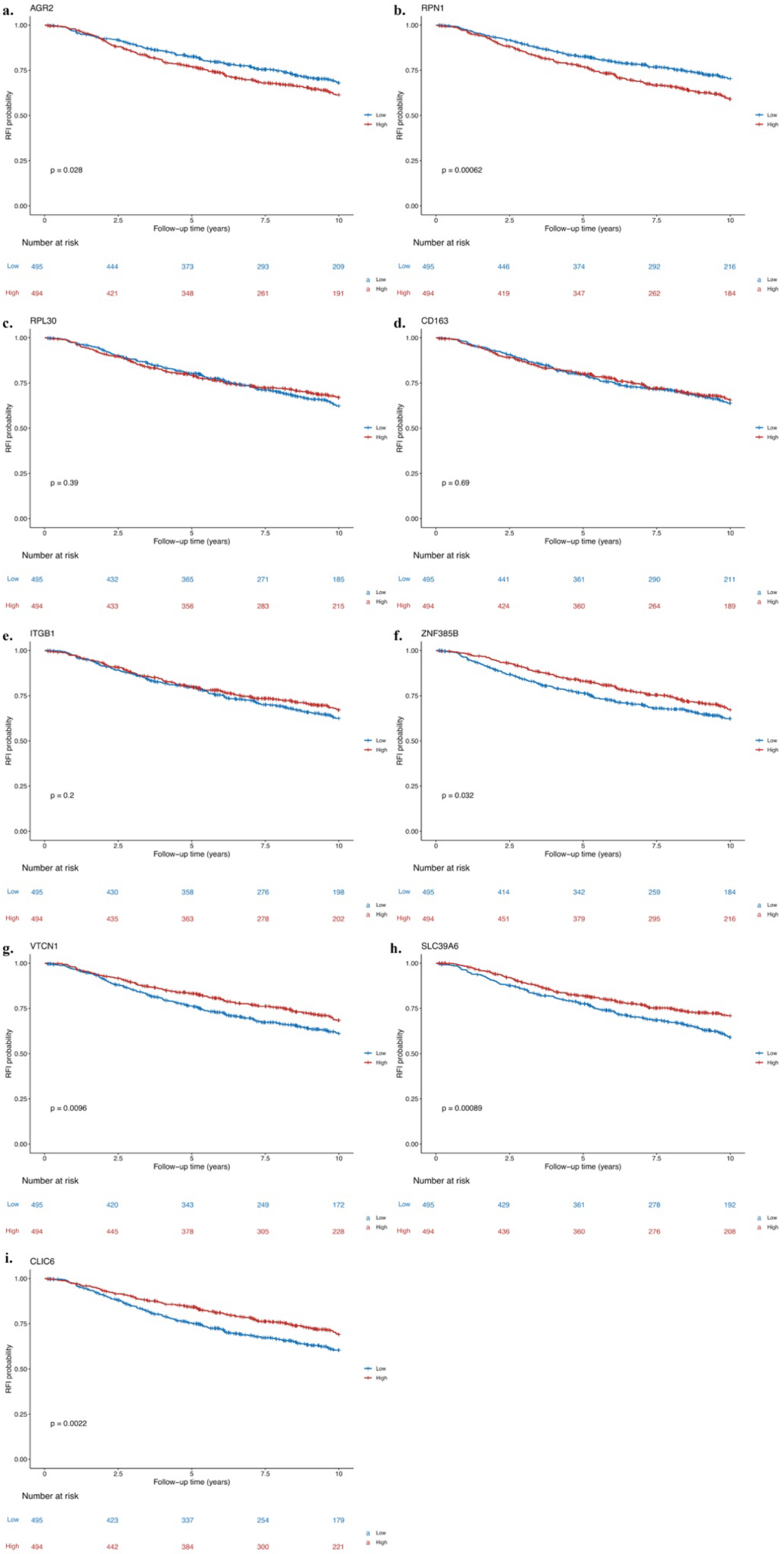

Supplementary Figure 6. Kaplan-Meier plots of the of the recurrent-free interval (RFI) of the METABRIC cohort comparing high (red) and low (blue) expression of the top 5 up-and downregulated genes (note, except *TRAJ14*, Entrez ID: 28741, not present in the METABRIC analysis) in the Endoresist cohorts' comparison of primary tumors of endocrine-resistant and endocrine-sensitive patients. Genes presented in order of highest to lowest linear fold change from the Endoresist analysis for RFI; *AGR2* (a), *RPN1* (b), *RPL30* (c), *CD163* (d), *ITGB1* (e), *ZNF385B* (f), *VTCN1* (g), *SLC39A6* (h), and *CLIC6* (i).

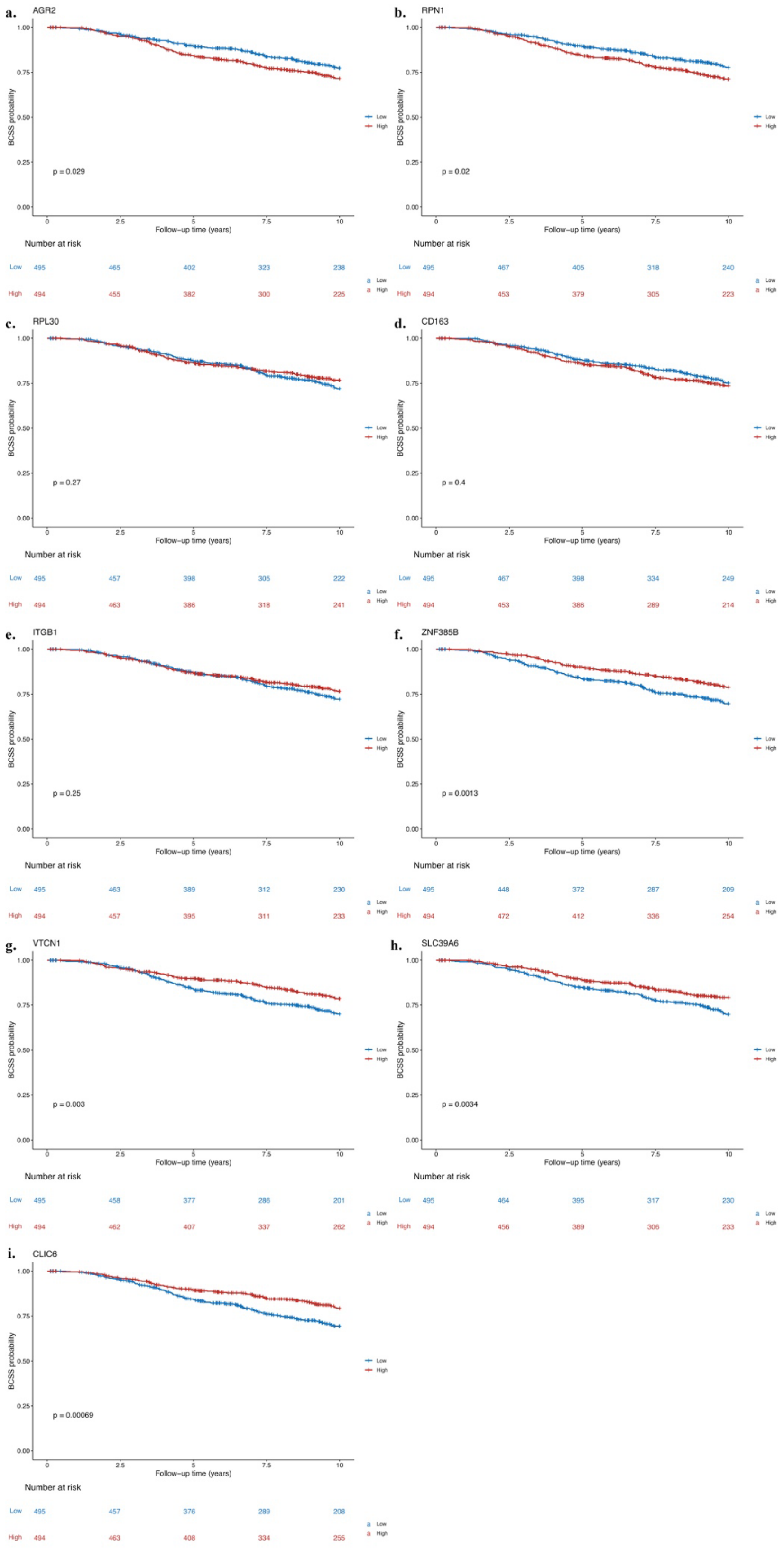

Supplementary Figure 7. Kaplan-Meier plots of the breast cancer-specific survival (BCSS) of the METABRIC cohort comparing high (red) and low (blue) expression of the top 5 up- and downregulated genes (note, except *TRAJ14*, Entrez ID: 28741, not present in the METABRIC analysis) in the Endoresist cohorts' comparison of primary tumors of endocrine-resistant and endocrine-sensitive patients. Genes presented in order of highest to lowest linear fold change from the Endoresist analysis for BCSS; *AGR2* (a), *RPN1* (b), *RPL30* (c), *CD163* (d), *ITGB1* (e), *ZNF385B* (f), *VTCN1* (g), *SLC39A6* (h), and *CLIC6* (i).

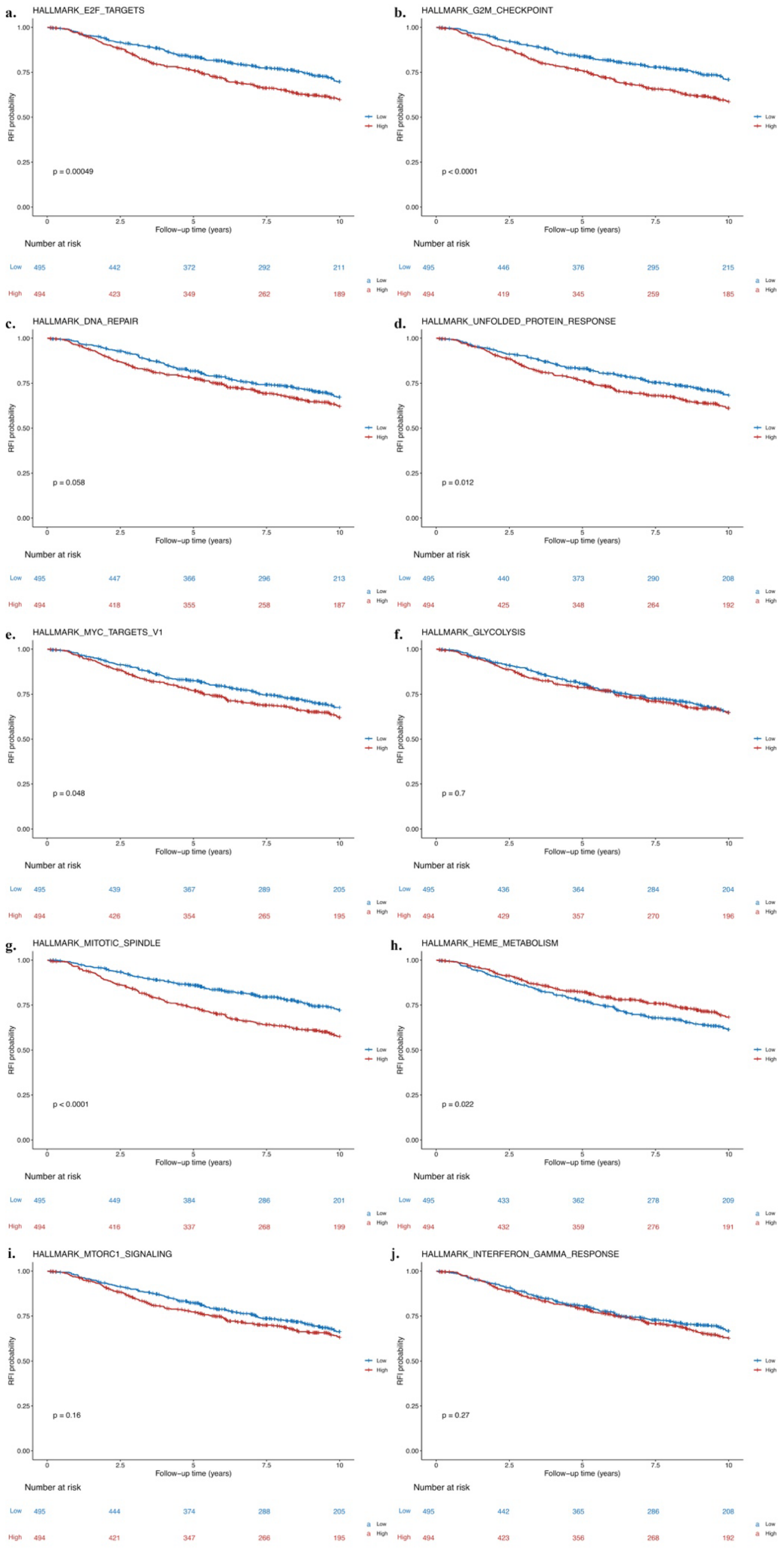

Supplementary Figure 8. Kaplan-Meier plots of the recurrent-free interval (RFI) of the METABRIC cohort comparing high (red) and low (blue) expression of the top 10 upregulated gene sets in the Endoresist cohorts' comparison of primary tumors of endocrine-resistant and endocrine-sensitive patients. Hallmark gene sets presented in order of highest net enrichment score from the Endoresist analysis for RFI; E2F_TARGETS (a), G2M_ CHECKPOINT (b), DNA_REPAIR (c), UNFOLDED_ PROTEIN_ RESPONSE (d), MYC_TARGETS _V1 (e), GLYCOLYSIS (f), MITOTIC_ SPINDLE (g), HEME_ METABOLISM (h), MTORC1_ SIGNALING (i), and INTERFERON_ GAMMA_ RESPONSE (j).

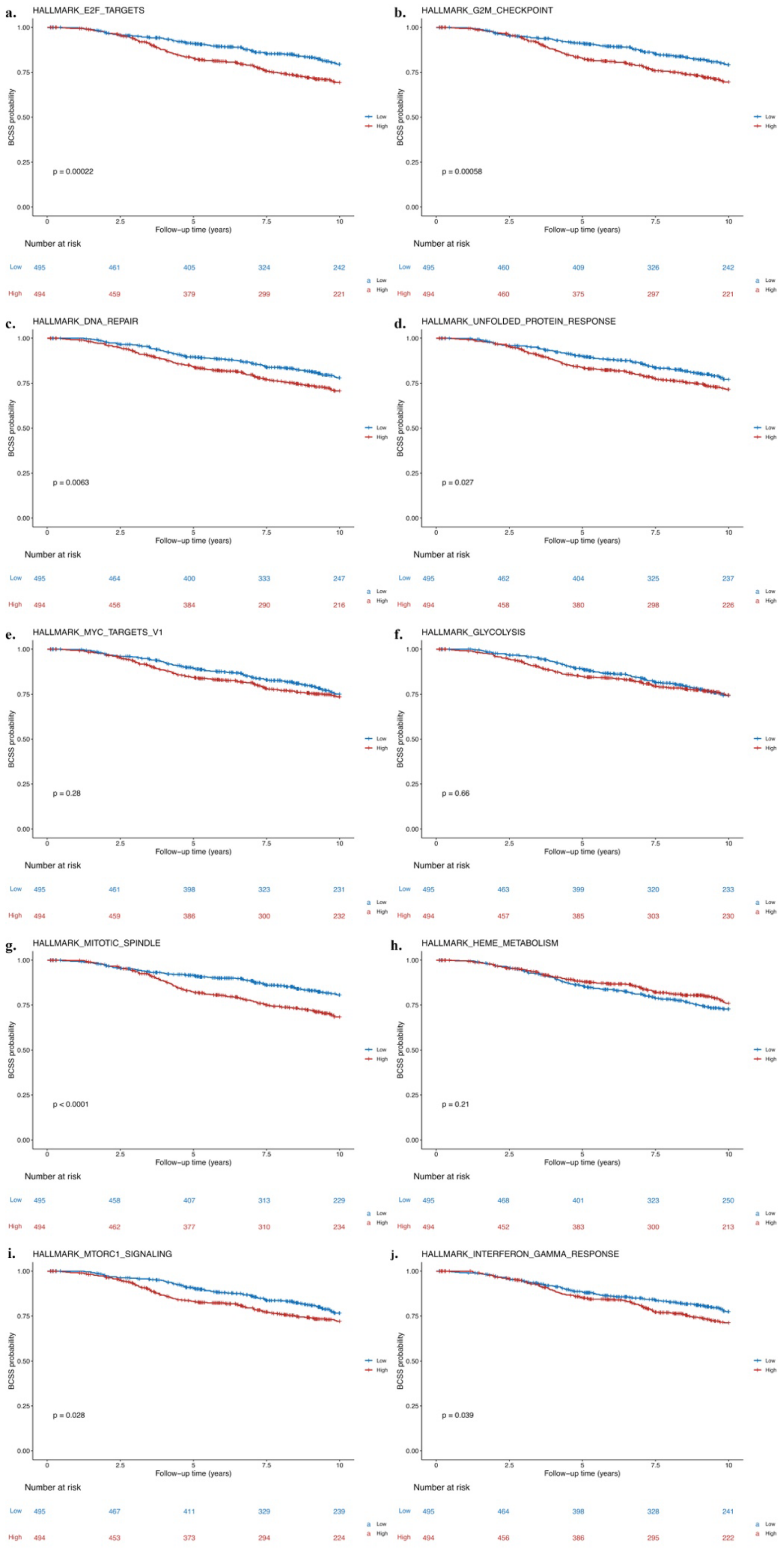

Supplementary Figure 9. Kaplan-Meier plots of the breast cancer-specific survival (BCSS) of the METABRIC cohort comparing high (red) and low (blue) expression of the top 10 upregulated gene sets in the Endoresist cohorts' comparison of primary tumors of endocrine-resistant and endocrine-sensitive patients. Hallmark gene sets presented in order of highest net enrichment score from the Endoresist analysis for BCSS; E2F_TARGETS (a), G2M_ CHECKPOINT (b), DNA_REPAIR (c), UNFOLDED_ PROTEIN_ RESPONSE (d), MYC_TARGETS_ V1 (e), GLYCOLYSIS (f), MITOTIC_ SPINDLE (g), HEME_ METABOLISM (h), MTORC1_ SIGNALING (i), and INTERFERON_ GAMMA_ RESPONSE (j).

Supplementary Table 1. Biospecimen Reporting for Improved Study Quality (BRISQ) report of the study.

**Data elements**	**Information**
Biospecimen type	Untreated primary and treated relapse tumor material.
Anatomical site	Breast and recurrent tumor locations (ipsilateral, contralateral, or distant metastasis).
Disease status of patients	Breast cancer patients +/- recurrent disease
Clinical characteristics of patients	Adjuvant treatment with endocrine treatment<br>+/- chemotherapy<br>+/- radiotherapy.
Vital state of patients	Alive at the time of tumor excision.
Clinical diagnosis of patients	Breast cancer
Pathology diagnosis	Invasive breast cancer
Collection mechanism	Surgical specimen and in a few cases core needle biopsy, formalin fixation.
Type of stabilization	None, room temperature
Type of long-term preservation	Formalin fixation
Constitution of preservative	10% formalin
Storage temperature	Room temperature
Storage duration	2-21 years
Shipping temperature	Room temperature
Composition, assessment & selection	Primary endocrine-treated ER-positive and HER2-negative tumors +/- ER-positive recurrent tumors. See materials and methods.

Supplementary Table 2. Clinicopathological characteristics of all primary and relapse tumors from patients with endocrine-resistant breast cancer (PERBC).

	PERBC (primary tumors) N=69	PERBC (relapse tumors) N=69	P-value PERBC relapse vs. primary tumors
**ER, %**			*p*=.83
Median: median (range), %	90 (5-100)	90 (20-100)	
**PR, %**			*p*=.0048
Median: median (range), %	50 (0-100)	7.5 (0-100)	
**PR status**			*p*=.011
Negative (<10%)	15 (21.74%)	29 (42.03%)	
Positive (≥10%)	39 (56.52%)	26 (37.68%)	
N/A	15 (21.74%)	14 (20.29%)	
**Ki67, %**			*p*=.39
Median: median (range), %	20 (1-95)	21.5 (0-100)	
**HER2 status**			*p*<.00001
HER2-negative/zero	42 (60.87%)	31 (44.93%)	
HER2-low	24 (34.78%)	31 (44.93%)	
HER2-positive	0 (0.00%)	1 (1.45%)	
N/A	3 (4.35%)	6 (8.70%)	
**TIL scoring**			*p*=.48
Median: median (range), %	5 (1-40)	7.5 (1-40)	

PERBC=patients with endocrine-resistant breast cancer, PESBC=patients with endocrine-sensitive breast cancer, ER=estrogen receptor, PR=progesterone receptor, TIL=tumor infiltrating lymphocyte, N/A=data not available.

Supplementary Table 3. Signature information of all patients in the Endoresist cohort, divided into tumor categories.

	PERBC (primary tumors) N=69	PESBC (primary tumors) N=77	Entire cohort (PERBC & PESBC primary tumors) N=146	P-value PERBC vs. PESBC primary tumors	PERBC (relapse tumors) N=69	P-value PERBC relapse vs. primary tumors
**PAM50 status**				*p*=.14		*p*=.85
Luminal A	34 (49.28%)	50 (64.94%)	84 (57.53%)		33 (47.83%)	
Luminal B	26 (37.68%)	23 (29.87%)	49 (33.56%)		22 (31.88%)	
HER2-enriched	3 (4.35%)	0 (0.00%)	3 (2.05%)		6 (8.70%)	
Basal	2 (2.90%)	1 (1.30%)	3 (2.05%)		3 (4.35%)	
Normal	1 (1.45%)	3 (3.90%)	4 (2.74%)		1 (1.45%)	
N/A	3 (4.35%)	0 (0.00%)	3 (2.05%)		4 (5.80%)	
**ROR-S group**				*p*=.0094		*p*=.23
Low	15 (21.74%)	35 (45.45%)	50 (34.25%)		10 (14.49%)	
Med	49 (71.01%)	39 (50.65%)	88 (60.27%)		49 (71.01%)	
High	2 (2.90%)	3 (3.90%)	5 (3.42%)		6 (8.70%)	
N/A	3 (4.35%)	0 (0.00%)	3 (2.05%)		4 (5.80%)	
**CES group**				*p*=.062		*p*=.20
CES-E	27 (39.13%)	46 (59.74%)	73 (50.00%)		18 (26.09%)	
CES-U	26 (37.68%)	23 (29.87%)	49 (33.56%)		27 (39.13%)	
CES-C	13 (18.84%)	8 (10.39%)	21 (14.38%)		20 (28.99%)	
N/A	3 (4.35%)	0 (0.00%)	3 (2.05%)		4 (5.80%)	

PERBC=patients with endocrine-resistant breast cancer, PESBC=patients with endocrine-sensitive breast cancer, cont.=continuous, PAM50=50 gene signature intrinsic subtype, ROR-S: risk of recurrence score based on intrinsic subtype, CES=chemoendocrine score, CES-E=endocrine responsive group, CES-U=uncertain group, CES-C=chemotherapy responsive group, N/A=data not available.

Supplementary Table 4. Gene set enrichment Analysis (GSEA) output of the Hallmark gene set comparisons, from the analysis of the merged cohorts' primary tumors of endocrine-resistant patients compared to endocrine-sensitive patients. Significant gene sets from the FDR q-value 0.10 cut-off.

NAME	NES	NOM p-val	FDR q-val
HALLMARK_E2F_TARGETS	1.73114	0.00607	0.08782
HALLMARK_G2M_CHECKPOINT	1.73027	0.00797	0.04501
HALLMARK_DNA_REPAIR	1.72494	0.01556	0.03320
HALLMARK_UNFOLDED_PROTEIN_RESPONSE	1.71349	0.00392	0.02906
HALLMARK_MYC_TARGETS_V1	1.70106	0.02140	0.02840
HALLMARK_GLYCOLYSIS	1.67593	0.01008	0.03483
HALLMARK_MITOTIC_SPINDLE	1.63968	0.02178	0.04789
HALLMARK_HEME_METABOLISM	1.63620	0.01010	0.04390
HALLMARK_MTORC1_SIGNALING	1.62496	0.02376	0.04466
HALLMARK_INTERFERON_GAMMA_RESPONSE	1.62403	0.02381	0.04040
HALLMARK_UV_RESPONSE_UP	1.59987	0.01544	0.05093
HALLMARK_REACTIVE_OXYGEN_SPECIES_PATHWAY	1.57932	0.02783	0.05693
HALLMARK_P53_PATHWAY	1.57652	0.00982	0.05430
HALLMARK_COMPLEMENT	1.54569	0.01804	0.07131
HALLMARK_PI3K_AKT_MTOR_SIGNALING	1.54177	0.04339	0.06979
HALLMARK_INTERFERON_ALPHA_RESPONSE	1.52158	0.07800	0.07799
HALLMARK_APICAL_JUNCTION	1.50666	0.04192	0.08168
HALLMARK_OXIDATIVE_PHOSPHORYLATION	1.49212	0.08135	0.08580
HALLMARK_SPERMATOGENESIS	1.48029	0.01207	0.08897
HALLMARK_PROTEIN_SECRETION	1.46881	0.06982	0.09194

NES=normalized enrichment score, nom p-value=nominal p-value, FDR q-value=false discovery rate p-value.

Supplementary Table 5a. Gene set enrichment Analysis (GSEA) output of the REACTOME gene set comparisons, from the analysis of the merged cohorts' primary tumors of endocrine-resistant patients compared to endocrine-sensitive patients. Significant gene sets from the FDR q-value 0.10 cut-off.

NAME	NES	NOM p-val	FDR q-val
REACTOME_TP53_REGULATES_METABOLIC_GENES	1.61124	0.01235	0.09990
REACTOME_DNA_METHYLATION	1.61072	0.04481	0.09968
REACTOME_TRANSCRIPTIONAL_AND_POST_TRANSLATIONAL_REGULATION_OF_MITF_M_EXPRESSION_AND_ACTIVITY	1.61064	0.00810	0.09902
REACTOME_PHOSPHORYLATION_OF_THE_APC_C	1.60990	0.03119	0.09901
REACTOME_ABERRANT_REGULATION_OF_MITOTIC_G1_S_TRANSITION_IN_CANCER_DUE_TO_RB1_DEFECTS	1.60897	0.02008	0.09927
REACTOME_RNA_POLYMERASE_II_TRANSCRIPTION_TERMINATION	1.60878	0.02429	0.09874
REACTOME_INTRINSIC_PATHWAY_FOR_APOPTOSIS	1.60775	0.02254	0.09898
REACTOME_VITAMIN_B5_PANTOTHENATE_METABOLISM	1.60775	0.01677	0.09832
REACTOME_TRANSCRIPTIONAL_REGULATION_BY_TP53	1.60709	0.01674	0.09836
REACTOME_ASPARAGINE_N_LINKED_GLYCOSYLATION	1.60603	0.02200	0.09889
REACTOME_PROGRAMMED_CELL_DEATH	1.60512	0.01653	0.09929
REACTOME_DISORDERS_OF_TRANSMEMBRANE_TRANSPORTERS	1.60508	0.01423	0.09875
REACTOME_MITOTIC_SPINDLE_CHECKPOINT	1.60507	0.01636	0.09812
REACTOME_ER_TO_GOLGI_ANTEROGRADE_TRANSPORT	1.60481	0.02626	0.09775
REACTOME_HOST_INTERACTIONS_OF_HIV_FACTORS	1.60474	0.03614	0.09717
REACTOME_TRANSCRIPTIONAL_REGULATION_BY_RUNX2	1.60452	0.02227	0.09678
REACTOME_MRNA_SPLICING	1.60400	0.03512	0.09672
REACTOME_GENE_SILENCING_BY_RNA	1.60363	0.03937	0.09632
REACTOME_INTERLEUKIN_7_SIGNALING	1.60168	0.01431	0.09754
REACTOME_INTERFERON_SIGNALING	1.60157	0.01663	0.09699
REACTOME_INSULIN_PROCESSING	1.60130	0.01829	0.09660
REACTOME_SARS_COV_2_INFECTION	1.59755	0.03099	0.09967
REACTOME_SIGNALING_BY_NOTCH	1.59740	0.01833	0.09919
REACTOME_METABOLISM_OF_AMINO_ACIDS_AND_DERIVATIVES	1.59716	0.03455	0.09876
REACTOME_RECRUITMENT_OF_MITOTIC_CENTROSOME_PROTEINS_AND_COMPLEXES	1.59619	0.02008	0.09902
REACTOME_SARS_COV_2_ACTIVATES_MODULATES_INNATE_AND_ADAPTIVE_IMMUNE_RESPONSES	1.59491	0.02088	0.09972
REACTOME_REGULATION_OF_RUNX2_EXPRESSION_AND_ACTIVITY	1.59415	0.03226	0.09972
REACTOME_TNF_SIGNALING	1.58973	0.04208	0.09999
REACTOME_ANTIVIRAL_MECHANISM_BY_IFN_STIMULATED_GENES	1.58924	0.02911	0.09981
REACTOME_HDACS_DEACETYLATE_HISTONES	1.58875	0.04637	0.09961
REACTOME_GOLGI_ASSOCIATED_VESICLE_BIOGENESIS	1.58604	0.02484	0.09986
REACTOME_NEDDYLATION	1.57863	0.01633	0.09983

REACTOME_NUCLEAR_PORE_COMPLEX_NPC_DISASSEMBLY	1.57863	0.03878	0.09932
REACTOME_SIALIC_ACID_METABOLISM	1.57808	0.01965	0.09921
REACTOME_TCF_DEPENDENT_SIGNALING_IN_RESPONSE_TO_WNT	1.57707	0.02911	0.09970
REACTOME_SIGNALING_BY_WNT	1.57665	0.01860	0.09964
REACTOME_SUMOYLATION_OF_UBIQUITINYLATION_PROTEINS	1.57631	0.02218	0.09947
REACTOME_CLATHRIN_MEDIATED_ENDOCYTOSIS	1.57596	0.03036	0.09932
REACTOME_PROTEIN_UBIQUITINATION	1.57577	0.03232	0.09912
REACTOME_NEF_MEDIATES_DOWN_MODULATION_OF_CELL_SURFACE_RECEPTORS_BY_RECRUITING_THEM_TO_CLATHRIN_ADAPTERS	1.57533	0.03607	0.09918
REACTOME_METABOLISM_OF_POLYAMINES	1.57508	0.05613	0.09887
REACTOME_REGULATION_OF_ENDOGENOUS_RETROELEMENTS_BY_PIWI_INTERACTING_RNAS_PIRNAS	1.57493	0.04427	0.09847
REACTOME_SEMA3A_PAK_DEPENDENT_AXON_REPULSION	1.57490	0.01633	0.09802
REACTOME_AURKA_ACTIVATION_BY_TPX2	1.57444	0.02434	0.09788
REACTOME_CYCLIN_D_ASSOCIATED_EVENTS_IN_G1	1.57382	0.02028	0.09788
REACTOME_PI_METABOLISM	1.57329	0.01429	0.09794
REACTOME_HOMOLOGOUS_DNA_PAIRING_AND_STRAND_EXCHANGE	1.57066	0.03571	0.09988
REACTOME_SOMITOGENESIS	1.57011	0.03549	0.09988
REACTOME_RRNA_PROCESSING	1.57010	0.06986	0.09941
REACTOME_COOPERATION_OF_PDCL_PHLP1_AND_TRIC_CCT_IN_G_PROTEIN_BETA_FOLDING	1.56975	0.02000	0.09927
REACTOME_KEAP1_NFE2L2_PATHWAY	1.56740	0.03862	0.09995
REACTOME_SUMOYLATION_OF_RNA_BINDING_PROTEINS	1.56734	0.03043	0.09954
REACTOME_POLYMERASE_SWITCHING_ON_THE_C_STRAND_OF_THE_TELOMERE	1.56729	0.03181	0.09910
REACTOME_RUNX1_REGULATES_GENES_INVOLVED_IN_MEGAKARYOCYTE_DIFFERENTIATION_AND_PLATELET_FUNCTION	1.56718	0.03043	0.09874
REACTOME_RMTS_METHYLATE_HISTONE_ARGININES	1.56679	0.04312	0.09864
REACTOME_RESOLUTION_OF_SISTER_CHROMATID_COHESION	1.56622	0.03462	0.09874
REACTOME_CELL_CELL_COMMUNICATION	1.56553	0.02464	0.09898
REACTOME_NUCLEAR_IMPORT_OF_REV_PROTEIN	1.56436	0.03049	0.09959
REACTOME_REGULATION_OF_PTEN_STABILITY_AND_ACTIVITY	1.56351	0.04527	0.09997
REACTOME_PROTEIN_FOLDING	1.55749	0.03893	0.09991
REACTOME_RAB_REGULATION_OF_TRAFFICKING	1.55721	0.03006	0.09974
REACTOME_TRANSPORT_OF_THE_SLBP_DEPENDANT_MATURE_MRNA	1.55675	0.04107	0.09968
REACTOME_REGULATION_OF_GLUCOKINASE_BY_GLUCOKINASE_REGULATORY_PROTEIN	1.55666	0.04303	0.09933
REACTOME_EPH_EPHRIN_SIGNALING	1.55590	0.03469	0.09962
REACTOME_INHIBITION_OF_DNA_RECOMBINATION_AT_TELOMERE	1.55403	0.04242	0.09988
REACTOME_TRANSCRIPTION_COUPLED_NUCLEOTIDE_EXCISION_REPAIR_TC_NER	1.54900	0.04694	0.09988

REACTOME_TRANSCRIPTIONAL_REGULATION_BY_RUNX3	1.54895	0.03659	0.09952
REACTOME_TAK1_DEPENDENT_IKK_AND_NF_KAPPA_B_ACTIVATION	1.54876	0.02459	0.09927
REACTOME_TRANSLOCATION_OF_SLC2A4_GLUT4_TO_THE_PLASMA_MEMBRANE	1.54755	0.04802	0.09972
REACTOME_TRANSCRIPTIONAL_REGULATION_BY_RUNX1	1.54675	0.03259	0.09972
REACTOME_APOPTOSIS	1.54547	0.03151	0.09986
REACTOME_NS1_MEDIATED_EFFECTS_ON_HOST_PATHWAYS	1.54437	0.03942	0.09998
REACTOME_E2F_MEDIATED_REGULATION_OF_DNA_REPLICATION	1.54413	0.00830	0.09984
REACTOME_SARS_COV_1_INFECTION	1.54376	0.08249	0.09982
REACTOME_LATE_SARS_COV_2_INFECTION_EVENTS	1.54371	0.04564	0.09948
REACTOME_RHO_GTPASE_CYCLE	1.54323	0.02686	0.09948
REACTOME_SLC_TRANSPORTER_DISORDERS	1.54069	0.01807	0.09970
REACTOME_HIV_LIFE_CYCLE	1.52525	0.04421	0.09996

NES=normalized enrichment score, nom p-value=nominal p-value, FDR q-value=false discovery rate p-value.

Supplementary Table 5b. Gene set enrichment Analysis (GSEA) output of the KEGG gene set comparisons, from the analysis of the merged cohorts' primary tumors of endocrine-resistant patients compared to endocrine-sensitive patients. Significant gene sets from the FDR q-value 0.10 cut-off.

NAME	NES	NOM p-val	FDR q-val
KEGG_VASOPRESSIN_REGULATED_WATER_REABSORPTION	1.82642	0.00203	0.08467
KEGG_SNARE_INTERACTIONS_IN_VESICULAR_TRANSPORT	1.82474	0.00000	0.05719
KEGG_CELL_CYCLE	1.79543	0.00000	0.05827
KEGG_SYSTEMIC_LUPUS_ERYTHEMATOSUS	1.68285	0.02088	0.09787
KEGG_GLIOMA	1.65852	0.00638	0.09979
KEGG_ENDOMETRIAL_CANCER	1.65260	0.00619	0.09877
KEGG_CHRONIC_MYELOID_LEUKEMIA	1.65073	0.00422	0.09386
KEGG_NUCLEOTIDE_EXCISION_REPAIR	1.62638	0.02075	0.09728
KEGG_PANTOTHENATE_AND_COA_BIOSYNTHESIS	1.62611	0.00787	0.09295
KEGG_BASAL_TRANSCRIPTION_FACTORS	1.62467	0.01035	0.08969
KEGG_GLYCOSAMINOGLYCAN_BIOSYNTHESIS_CHONDROITIN_SU	1.62331	0.02335	0.08669
KEGG_PROSTATE_CANCER	1.61930	0.01064	0.08580
KEGG_MISMATCH_REPAIR	1.60355	0.02505	0.09572
KEGG_ANTIGEN_PROCESSING_AND_PRESENTATION	1.59728	0.03354	0.09721
KEGG_AMINOACYL_TRNA_BIOSYNTHESIS	1.57215	0.03259	0.09886
KEGG_FC_GAMMA_R_MEDIATED_PHAGOCYTOSIS	1.57175	0.02088	0.09605
KEGG_NOTCH_SIGNALING_PATHWAY	1.57110	0.02766	0.09400
KEGG_PARKINSONS_DISEASE	1.55988	0.05155	0.09799
KEGG_ARGININE_AND_PROLINE_METABOLISM	1.55951	0.02282	0.09546
KEGG_ADHERENS_JUNCTION	1.55574	0.03942	0.09564
KEGG_T_CELL_RECEPTOR_SIGNALING_PATHWAY	1.55235	0.02079	0.09566
KEGG_NOD_LIKE_RECEPTOR_SIGNALING_PATHWAY	1.55216	0.02259	0.09335

KEGG_ENDOCYTOSIS	1.54303	0.03498	0.09876
KEGG_PYRUVATE_METABOLISM	1.54078	0.04082	0.09836
KEGG_PRION_DISEASES	1.53932	0.02590	0.09746
KEGG_FRUCTOSE_AND_MANNOSE_METABOLISM	1.52357	0.05010	0.09939
KEGG_PURINE_METABOLISM	1.52314	0.03093	0.09772
KEGG_TIGHT_JUNCTION	1.52048	0.04409	0.09813

NES=normalized enrichment score, nom p-value=nominal p-value, FDR q-value=false discovery rate p-value.

Supplementary Table 6a. Gene set enrichment Analysis (GSEA) output of the Hallmark gene set comparisons, from the analysis of the merged cohorts' endocrine-resistant patients relapse and primary tumors, using the preranked GSEA with the primary ranking metric (–$\log_{10}$(p-value)). Significant gene sets from the FDR q-value 0.10 cut-off.

NAME	NES	NOM p-val	FDR q-val
HALLMARK_XENOBIOTIC_METABOLISM	2.29986	0.00000	0.00000
HALLMARK_COAGULATION	1.95162	0.00000	0.00000
HALLMARK_CHOLESTEROL_HOMEOSTASIS	1.83521	0.00000	0.00070
HALLMARK_PEROXISOME	1.83164	0.00000	0.00053
HALLMARK_BILE_ACID_METABOLISM	1.82294	0.00000	0.00042
HALLMARK_FATTY_ACID_METABOLISM	1.64891	0.00000	0.00759
HALLMARK_OXIDATIVE_PHOSPHORYLATION	1.63446	0.00000	0.00775
HALLMARK_ADIPOGENESIS	1.61279	0.00000	0.00697
HALLMARK_COMPLEMENT	1.54762	0.00000	0.01029
HALLMARK_ESTROGEN_RESPONSE_LATE	-1.39176	0.01063	0.09614
HALLMARK_MITOTIC_SPINDLE	-1.54715	0.00115	0.01601
HALLMARK_TNFA_SIGNALING_VIA_NFKB	-1.56006	0.00000	0.01694
HALLMARK_MYC_TARGETS_V1	-1.57732	0.00000	0.01687
HALLMARK_G2M_CHECKPOINT	-1.61763	0.00000	0.01303
HALLMARK_E2F_TARGETS	-1.65904	0.00000	0.01048
HALLMARK_ESTROGEN_RESPONSE_EARLY	-1.93909	0.00000	0.00000

NES=normalized enrichment score, nom p-value=nominal p-value, FDR q-value=false discovery rate p-value.

Supplementary Table 6b. Gene set enrichment Analysis (GSEA) output of the Hallmark gene set comparisons, from the analysis of the merged cohorts' endocrine-resistant patients relapse and primary tumors, using the preranked GSEA with the secondary ranking metric ($\log_2$FC). This analysis emphasizes effect size, whereas the primary metric center on statistical confidence combined with directionality. Significant gene sets from the FDR q-value 0.10 cut-off.

NAME	NES	NOM p-val	FDR q-val
HALLMARK_OXIDATIVE_PHOSPHORYLATION	1.96584	0.00000	0.00240
HALLMARK_COMPLEMENT	1.90272	0.00000	0.00508
HALLMARK_CHOLESTEROL_HOMEOSTASIS	1.86107	0.00000	0.00339
HALLMARK_XENOBIOTIC_METABOLISM	1.85295	0.00000	0.00294
HALLMARK_COAGULATION	1.69935	0.00820	0.00727
HALLMARK_PEROXISOME	1.68164	0.00388	0.00841
HALLMARK_BILE_ACID_METABOLISM	1.62710	0.00000	0.01232
HALLMARK_FATTY_ACID_METABOLISM	1.61357	0.00446	0.01232

HALLMARK_ADIPOGENESIS	1.59487	0.00000	0.01265
HALLMARK_TGF_BETA_SIGNALING	1.50127	0.01294	0.02316
HALLMARK_INTERFERON_GAMMA_RESPONSE	1.47077	0.00000	0.02763
HALLMARK_PROTEIN_SECRETION	1.46439	0.00400	0.02718
HALLMARK_APOPTOSIS	1.41868	0.00000	0.03845
HALLMARK_IL6_JAK_STAT3_SIGNALING	1.35119	0.02439	0.06314
HALLMARK_KRAS_SIGNALING_UP	1.34092	0.02186	0.06490
HALLMARK_INTERFERON_ALPHA_RESPONSE	1.30578	0.04511	0.08214
HALLMARK_IL2_STAT5_SIGNALING	1.28525	0.03333	0.08936
HALLMARK_TNFA_SIGNALING_VIA_NFKB	-1.42740	0.00607	0.06268
HALLMARK_UV_RESPONSE_DN	-1.43632	0.01170	0.06424
HALLMARK_E2F_TARGETS	-1.57977	0.00243	0.01613
HALLMARK_MITOTIC_SPINDLE	-1.60159	0.00000	0.01458
HALLMARK_ESTROGEN_RESPONSE_LATE	-1.61042	0.00000	0.01623
HALLMARK_G2M_CHECKPOINT	-1.71866	0.00000	0.00432
HALLMARK_MYC_TARGETS_V1	-1.83059	0.00000	0.00242
HALLMARK_ESTROGEN_RESPONSE_EARLY	-2.09249	0.00000	0.00000

NES=normalized enrichment score, nom p-value=nominal p-value, FDR q-value=false discovery rate p-value

Supplementary Table 7a. Gene set enrichment Analysis (GSEA) output of the REACTOME gene set comparisons, from the analysis of the merged cohorts' endocrine-resistant patients relapse and primary tumors. Significant gene sets from the FDR q-value 0.10 cut-off.

**NAME**	**NES**	**NOM p-val**	**FDR q-val**
REACTOME_SYNTHESIS_OF_BILE_ACIDS_AND_BILE_SALTS_VIA_7ALPHA_HYDROXYCHOLESTEROL	2.28306	0.00000	0.00000
REACTOME_ASPIRIN_ADME	2.22799	0.00000	0.00054
REACTOME_REGULATION_OF_TLR_BY_ENDOGENOUS_LIGAND	2.21372	0.00000	0.00077
REACTOME_MOLECULES_ASSOCIATED_WITH_ELASTIC_FIBRES	2.15007	0.00000	0.00058
REACTOME_FORMATION_OF_FIBRIN_CLOT_CLOTTING_CASCADE	2.13290	0.00000	0.00144
REACTOME_ELASTIC_FIBRE_FORMATION	2.12588	0.00000	0.00139
REACTOME_PLASMA_LIPOPROTEIN_ASSEMBLY	2.09096	0.00276	0.00242
REACTOME_BIOLOGICAL_OXIDATIONS	2.08877	0.00000	0.00212
REACTOME_RA_BIOSYNTHESIS_PATHWAY	2.08868	0.00000	0.00188
REACTOME_DRUG_ADME	2.06142	0.00000	0.00265
REACTOME_BILE_ACID_AND_BILE_SALT_METABOLISM	2.03573	0.00000	0.00350
REACTOME_PLASMA_LIPOPROTEIN_ASSEMBLY_REMODELING_AND_CLEARANCE	2.00938	0.00000	0.00513
REACTOME_PHASE_II_CONJUGATION_OF_COMPOUNDS	1.98708	0.00000	0.00632
REACTOME_INTRINSIC_PATHWAY_OF_FIBRIN_CLOT_FORMATION	1.96838	0.00287	0.00746
REACTOME_COMPLEMENT_CASCADE	1.96750	0.00000	0.00696
REACTOME_PHASE_I_FUNCTIONALIZATION_OF_COMPOUNDS	1.96331	0.00000	0.00668
REACTOME_COMMON_PATHWAY_OF_FIBRIN_CLOT_FORMATION	1.96251	0.00000	0.00636
REACTOME_SIGNAL_TRANSDUCTION_BY_L1	1.95963	0.00000	0.00641
REACTOME_RECYCLING_OF_BILE_ACIDS_AND_SALTS	1.90514	0.00000	0.01326
REACTOME_METABOLISM_OF_FAT_SOLUBLE_VITAMINS	1.89528	0.00000	0.01478
REACTOME_KERATAN_SULFATE_DEGRADATION	1.85567	0.00000	0.02240

REACTOME_RESPONSE_TO_ELEVATED_PLATELET_CYTOSOLIC_CA2	1.85438	0.00000	0.02166
REACTOME_N_GLYCAN_TRIMMING_IN_THE_ER_AND_CALNEXIN_CALRETICULIN_CYCLE	1.85417	0.00000	0.02082
REACTOME_METABOLISM_OF_PORPHYRINS	1.81605	0.00000	0.02997
REACTOME_DETOXIFICATION_OF_REACTIVE_OXYGEN_SPECIES	1.81442	0.00000	0.02926
REACTOME_ANTIMICROBIAL_PEPTIDES	1.80800	0.00426	0.03039
REACTOME_METABOLISM_OF_STEROIDS	1.79243	0.00000	0.03451
REACTOME_VISUAL_PHOTOTRANSDUCTION	1.76334	0.00000	0.04488
REACTOME_CALNEXIN_CALRETICULIN_CYCLE	1.74701	0.00000	0.05053
REACTOME_SYNTHESIS_OF_BILE_ACIDS_AND_BILE_SALTS	1.73964	0.00000	0.05287
REACTOME_DISEASES_OF_CARBOHYDRATE_METABOLISM	1.73587	0.00000	0.05270
REACTOME_THE_CANONICAL_RETINOID_CYCLE_IN_RODS_TWILIGHT_VISION	1.73569	0.00282	0.05113
REACTOME_REGULATION_OF_INSULIN_LIKE_GROWTH_FACTOR_IGF_TRANSPORT_AND_UPTAKE_BY_INSULIN_LIKE_GROWTH_FACTOR_BINDING_PROTEINS_IGFBPS	1.71139	0.00000	0.06256
REACTOME_HYALURONAN_METABOLISM	1.70721	0.02192	0.06307
REACTOME_IRAK4_DEFICIENCY_TLR2_4	1.70490	0.01058	0.06284
REACTOME_ION_CHANNEL_TRANSPORT	1.68528	0.00000	0.07309
REACTOME_GLUCURONIDATION	1.68370	0.00279	0.07197
REACTOME_FATTY_ACID_METABOLISM	1.67042	0.00000	0.07977
REACTOME_TRP_CHANNELS	1.66724	0.00595	0.07970
REACTOME_MITOCHONDRIAL_PROTEIN_DEGRADATION	1.66377	0.00000	0.08034
REACTOME_SIGNALING_BY_RETINOIC_ACID	1.65684	0.01000	0.08340
REACTOME_CYTOPROTECTION_BY_HMOX1	1.64629	0.00000	0.08826
REACTOME_HEME_SIGNALING	1.64471	0.00336	0.08723
REACTOME_NEUTROPHIL_DEGRANULATION	1.63965	0.00000	0.08827
REACTOME_EFFECTS_OF_PIP2_HYDROLYSIS	1.62869	0.01166	0.09386
REACTOME_PLASMA_LIPOPROTEIN_REMODELING	1.62555	0.01329	0.09432
REACTOME_CYTOSOLIC_SULFONATION_OF_SMALL_MOLECULES	1.61902	0.00890	0.09696
REACTOME_PROCESSING_OF_CAPPED_INTRON_CONTAINING_PRE_MRNA	-1.54140	0.00000	0.09302
REACTOME_PI_METABOLISM	-1.54510	0.01051	0.09064
REACTOME_INTERACTIONS_OF_VPR_WITH_HOST_CELLULAR_PROTEINS	-1.54622	0.01583	0.09042
REACTOME_CILIUM_ASSEMBLY	-1.55147	0.00000	0.08703
REACTOME_RESOLUTION_OF_ABASIC_SITES_AP_SITES	-1.55159	0.01360	0.08769
REACTOME_TRANSPORT_OF_MATURE_TRANSCRIPT_TO_CYTOPLASM	-1.55189	0.00679	0.08819
REACTOME_APC_CDC20_MEDIATED_DEGRADATION_OF_NEK2A	-1.55199	0.01644	0.08888
REACTOME_ABERRANT_REGULATION_OF_MITOTIC_EXIT_IN_CANCER_DUE_TO_RB1_DEFECTS	-1.55599	0.01983	0.08598
REACTOME_REGULATION_OF_TP53_ACTIVITY	-1.55726	0.00121	0.08552
REACTOME_APC_C_CDC20_MEDIATED_DEGRADATION_OF_CYCLIN_B	-1.55730	0.03514	0.08627
REACTOME_VEGFR2_MEDIATED_VASCULAR_PERMEABILITY	-1.55976	0.02757	0.08502
REACTOME_SUMOYLATION_OF_UBIQUITINYLATION_PROTEINS	-1.56054	0.01031	0.08512
REACTOME_SUMOYLATION_OF_SUMOYLATION_PROTEINS	-1.57104	0.01429	0.07766
REACTOME_INTERLEUKIN_RECEPTOR_SHC_SIGNALING	-1.57435	0.01815	0.07577
REACTOME_SIGNALING_BY_NUCLEAR_RECEPTORS	-1.57690	0.00000	0.07463

REACTOME_REGULATION_OF_GLUCOKINASE_BY_GLUCOKINASE_REGULATORY_PROTEIN	-1.57929	0.01773	0.07350
REACTOME_RNA_POLYMERASE_II_TRANSCRIPTION_TERMINATION	-1.58744	0.01364	0.06862
REACTOME_SIGNALING_BY_NTRK3_TRKC	-1.59281	0.02167	0.06576
REACTOME_ANCHORING_OF_THE_BASAL_BODY_TO_THE_PLASMA_MEMBRANE	-1.59755	0.00131	0.06327
REACTOME_EXTRA_NUCLEAR_ESTROGEN_SIGNALING	-1.59806	0.00793	0.06364
REACTOME_EXPORT_OF_VIRAL_RIBONUCLEOPROTEINS_FROM_NUCLEUS	-1.59814	0.01449	0.06421
REACTOME_M_PHASE	-1.60026	0.00000	0.06349
REACTOME_CHROMOSOME_MAINTENANCE	-1.60045	0.00000	0.06401
REACTOME_EUKARYOTIC_TRANSLATION_INITIATION	-1.60064	0.00365	0.06456
REACTOME_TRANSPORT_OF_THE_SLBP_DEPENDANT_MATURE_MRNA	-1.60521	0.01045	0.06209
REACTOME_PRE_NOTCH_EXPRESSION_AND_PROCESSING	-1.60529	0.00386	0.06268
REACTOME_HDMS_DEMETHYLATE_HISTONES	-1.60670	0.01149	0.06234
REACTOME_RRNA_PROCESSING	-1.60986	0.00000	0.06100
REACTOME_RHOBTB_GTPASE_CYCLE	-1.61134	0.00875	0.06063
REACTOME_ACTIVATION_OF_THE_PRE_REPLICATIVE_COMPLEX	-1.61714	0.00732	0.05732
REACTOME_DISEASES_OF_MITOTIC_CELL_CYCLE	-1.62078	0.00978	0.05582
REACTOME_TELOMERE_EXTENSION_BY_TELOMERASE	-1.62106	0.00939	0.05635
REACTOME_ACTIVATION_OF_ANTERIOR_HOX_GENES_IN_HINDBRAIN_DEVELOPMENT_DURING_EARLY_EMBRYOGENESIS	-1.62123	0.00127	0.05689
REACTOME_TRANSCRIPTIONAL_REGULATION_BY_RUNX1	-1.62307	0.00000	0.05651
REACTOME_ANTIGEN_ACTIVATES_B_CELL_RECEPTOR_BCR_LEADING_TO_GENERATION_OF_SECOND_MESSENGERS	-1.62394	0.00423	0.05677
REACTOME_PKMTS_METHYLATE_HISTONE_LYSINES	-1.62509	0.00556	0.05681
REACTOME_FCERI_MEDIATED_MAPK_ACTIVATION	-1.62908	0.00408	0.05557
REACTOME_CD28_DEPENDENT_PI3K_AKT_SIGNALING	-1.63472	0.01002	0.05308
REACTOME_RUNX1_INTERACTS_WITH_CO_FACTORS_WHOSE_PRECISE_EFFECT_ON_RUNX1_TARGETS_IS_NOT_KNOWN	-1.64070	0.01471	0.05034
REACTOME_ISG15_ANTIVIRAL_MECHANISM	-1.64114	0.00135	0.05069
REACTOME_SUMOYLATION_OF_CHROMATIN_ORGANIZATION_PROTEINS	-1.64427	0.00000	0.04958
REACTOME_SUMOYLATION_OF_DNA_DAMAGE_RESPONSE_AND_REPAIR_PROTEINS	-1.64520	0.00135	0.04973
REACTOME_SYNTHESIS_OF_PIPS_AT_THE_PLASMA_MEMBRANE	-1.64855	0.00420	0.04849
REACTOME_SUMOYLATION	-1.66873	0.00000	0.03931
REACTOME_RNA_POLYMERASE_I_PROMOTER_ESCAPE	-1.66987	0.00000	0.03930
REACTOME_INTERACTIONS_OF_REV_WITH_HOST_CELLULAR_PROTEINS	-1.67122	0.00439	0.03918
REACTOME_SARS_COV_1_MODULATES_HOST_TRANSLATION_MACHINERY	-1.67887	0.00145	0.03637
REACTOME_CONVERSION_FROM_APC_C_CDC20_TO_APC_C_CDH1_IN_LATE_ANAPHASE	-1.68124	0.00486	0.03585
REACTOME_SARS_COV_2_MODULATES_HOST_TRANSLATION_MACHINERY	-1.68558	0.00433	0.03458
REACTOME_RNA_POLYMERASE_I_TRANSCRIPTION	-1.69187	0.00000	0.03245
REACTOME_NUCLEAR_IMPORT_OF_REV_PROTEIN	-1.69213	0.00160	0.03287
REACTOME_SIGNALING_BY_FGFR1_IN_DISEASE	-1.69245	0.00149	0.03326
REACTOME_B_WICH_COMPLEX_POSITIVELY_REGULATES_RRNA_EXPRESSION	-1.69844	0.00000	0.03174
REACTOME_REGULATION_OF_MITF_M_DEPENDENT_GENES_INVOLVED_IN_PIGMENTATION	-1.70275	0.00287	0.03050

REACTOME_TRANSCRIPTIONAL_REGULATION_OF_GRANULOPOIESIS	-1.70445	0.00000	0.03033
REACTOME_RHO_GTPASES_ACTIVATE_PKNS	-1.70683	0.00000	0.03014
REACTOME_PHOSPHORYLATION_OF_THE_APC_C	-1.70686	0.00153	0.03062
REACTOME_TELOMERE_MAINTENANCE	-1.70789	0.00000	0.03075
REACTOME_RHOBTB1_GTPASE_CYCLE	-1.71774	0.00302	0.02734
REACTOME_REGULATION_OF_PTEN_GENE_TRANSCRIPTION	-1.72028	0.00277	0.02678
REACTOME_COSTIMULATION_BY_THE_CD28_FAMILY	-1.72413	0.00135	0.02594
REACTOME_ACTIVATION_OF_THE_MRNA_UPON_BINDING_OF_THE_CAP_BINDING_COMPLEX_AND_EIFS_AND_SUBSEQUENT_BINDING_TO_43S	-1.73850	0.00000	0.02206
REACTOME_SUMOYLATION_OF_RNA_BINDING_PROTEINS	-1.74111	0.00000	0.02181
REACTOME_DISEASES_OF_PROGRAMMED_CELL_DEATH	-1.74137	0.00000	0.02210
REACTOME_INHIBITION_OF_THE_PROTEOLYTIC_ACTIVITY_OF_APC_C_REQUIRED_FOR_THE_ONSET_OF_ANAPHASE_BY_MITOTIC_SPINDLE_CHECKPOINT_COMPONENTS	-1.74155	0.00160	0.02251
REACTOME_FGFR1_MUTANT_RECEPTOR_ACTIVATION	-1.75037	0.00156	0.02032
REACTOME_GENE_SILENCING_BY_RNA	-1.75134	0.00000	0.02041
REACTOME_NONSENSE_MEDIATED_DECAY_NMD	-1.75459	0.00000	0.01972
REACTOME_POSITIVE_EPIGENETIC_REGULATION_OF_RRNA_EXPRESSION	-1.75644	0.00000	0.01976
REACTOME_SYNTHESIS_OF_PIPS_AT_THE_EARLY_ENDOSOME_MEMBRANE	-1.75687	0.00312	0.02005
REACTOME_MITOTIC_PROPHASE	-1.76138	0.00000	0.01923
REACTOME_DAP12_SIGNALING	-1.77937	0.00303	0.01521
REACTOME_INHIBITION_OF_DNA_RECOMBINATION_AT_TELOMERE	-1.78254	0.00145	0.01476
REACTOME_DEPOSITION_OF_NEW_CENPA_CONTAINING_NUCLEOSOMES_AT_THE_CENTROMERE	-1.79624	0.00000	0.01248
REACTOME_NEGATIVE_EPIGENETIC_REGULATION_OF_RRNA_EXPRESSION	-1.79734	0.00000	0.01251
REACTOME_HCMV_INFECTION	-1.80703	0.00000	0.01111
REACTOME_HATS_ACETYLATE_HISTONES	-1.80805	0.00000	0.01115
REACTOME_TRANSCRIPTIONAL_REGULATION_BY_VENTX	-1.81130	0.00295	0.01092
REACTOME_DNA_REPLICATION	-1.81775	0.00000	0.01010
REACTOME_CONDENSATION_OF_PROPHASE_CHROMOSOMES	-1.82703	0.00000	0.00898
REACTOME_TRANSCRIPTIONAL_REGULATION_BY_SMALL_RNAS	-1.83057	0.00000	0.00884
REACTOME_HCMV_EARLY_EVENTS	-1.83256	0.00000	0.00879
REACTOME_OXIDATIVE_STRESS_INDUCED_SENESCENCE	-1.83693	0.00000	0.00834
REACTOME_EPIGENETIC_REGULATION_OF_GENE_EXPRESSION	-1.83957	0.00000	0.00824
REACTOME_ESR_MEDIATED_SIGNALING	-1.84023	0.00000	0.00840
REACTOME_DNA_REPLICATION_PRE_INITIATION	-1.84390	0.00000	0.00843
REACTOME_FORMATION_OF_THE_BETA_CATENIN_TCF_TRANSACTIVATING_COMPLEX	-1.84526	0.00000	0.00858
REACTOME_ESTROGEN_DEPENDENT_GENE_EXPRESSION	-1.84617	0.00000	0.00870
REACTOME_REPLACEMENT_OF_PROTAMINES_BY_NUCLEOSOMES_IN_THE_MALE_PRONUCLEUS	-1.85032	0.00000	0.00831
REACTOME_TRANSCRIPTIONAL_REGULATION_BY_E2F6	-1.85114	0.00000	0.00846
REACTOME_CD28_CO_STIMULATION	-1.85284	0.00000	0.00863
REACTOME_RUNX1_REGULATES_GENES_INVOLVED_IN_MEGAKARYOCYTE_DIFFERENTIATION_AND_PLATELET_FUNCTION	-1.87927	0.00000	0.00543
REACTOME_MEIOSIS	-1.88834	0.00000	0.00472
REACTOME_MEIOTIC_SYNAPSIS	-1.89000	0.00000	0.00480

REACTOME_REGULATION_OF_ENDOGENOUS_RETROELEMENTS	-1.89370	0.00000	0.00458
REACTOME_REGULATION_OF_ENDOGENOUS_RETROELEMENTS_BY_THE_HUMAN_SILENCING_HUB_HUSH_COMPLEX	-1.89507	0.00000	0.00477
REACTOME_SIGNALING_BY_CYTOSOLIC_FGFR1_FUSION_MUTANTS	-1.91155	0.00000	0.00348
REACTOME_MATERNAL_TO_ZYGOTIC_TRANSITION_MZT	-1.92027	0.00000	0.00310
REACTOME_REPRODUCTION	-1.93667	0.00000	0.00229
REACTOME_DNA_METHYLATION	-1.93710	0.00000	0.00241
REACTOME_SIRT1_NEGATIVELY_REGULATES_RRNA_EXPRESSION	-1.94146	0.00000	0.00220
REACTOME_REGULATION_OF_ENDOGENOUS_RETROELEMENTS_BY_PIWI_INTERACTING_RNAS_PIRNAS	-1.94913	0.00000	0.00202
REACTOME_ACTIVATED_PKN1_STIMULATES_TRANSCRIPTION_OF_AR_ANDROGEN_RECEPTOR_REGULATED_GENES_KLK2_AND_KLK3	-1.95010	0.00000	0.00214
REACTOME_CELLULAR_SENESCENCE	-1.95277	0.00000	0.00215
REACTOME_RMTS_METHYLATE_HISTONE_ARGININES	-1.95363	0.00000	0.00230
REACTOME_SENESCENCE_ASSOCIATED_SECRETORY_PHENOTYPE_SASP	-1.96136	0.00000	0.00201
REACTOME_REGULATION_OF_ENDOGENOUS_RETROELEMENTS_BY_KRAB_ZFP_PROTEINS	-1.96191	0.00000	0.00216
REACTOME_MEIOTIC_RECOMBINATION	-1.96892	0.00000	0.00227
REACTOME_HCMV_LATE_EVENTS	-1.98092	0.00000	0.00173
REACTOME_ERCC6_CSB_AND_EHMT2_G9A_POSITIVELY_REGULATE_RRNA_EXPRESSION	-1.98388	0.00000	0.00172
REACTOME_CHROMATIN_MODIFYING_ENZYMES	-1.98915	0.00000	0.00181
REACTOME_ASSEMBLY_OF_THE_ORC_COMPLEX_AT_THE_ORIGIN_OF_REPLICATION	-1.99777	0.00000	0.00170
REACTOME_BASE_EXCISION_REPAIR	-2.00623	0.00000	0.00169
REACTOME_BASE_EXCISION_REPAIR_AP_SITE_FORMATION	-2.01825	0.00000	0.00136
REACTOME_RECOGNITION_AND_ASSOCIATION_OF_DNA_GLYCOSYLASE_WITH_SITE_CONTAINING_AN_AFFECTED_PURINE	-2.04410	0.00000	0.00073
REACTOME_DNA_DAMAGE_TELOMERE_STRESS_INDUCED_SENESCENCE	-2.04459	0.00000	0.00091
REACTOME_PRC2_METHYLATES_HISTONES_AND_DNA	-2.05830	0.00000	0.00122
REACTOME_HDACS_DEACETYLATE_HISTONES	-2.06057	0.00000	0.00182
REACTOME_CHROMATIN_MODIFICATIONS_DURING_THE_MATERNAL_TO_ZYGOTIC_TRANSITION_MZT	-2.09613	0.00000	0.00181

NES=normalized enrichment score, nom p-value=nominal p-value, FDR q-value=false discovery rate p-value.

Supplementary Table 7b. Gene set enrichment Analysis (GSEA) output of the KEGG gene set comparisons, from the analysis of the merged cohorts' endocrine-resistant patients relapse and primary tumors. Significant gene sets from the FDR q-value 0.10 cut-off.

NAME	NES	NOM p-val	FDR q-val
KEGG_COMPLEMENT_AND_COAGULATION_CASCADES	2.43792	0.00000	0.00000
KEGG_METABOLISM_OF_XENOBIOTICS_BY_CYTOCHROME_P450	2.36915	0.00000	0.00000
KEGG_DRUG_METABOLISM_CYTOCHROME_P450	2.25950	0.00000	0.00082
KEGG_TRYPTOPHAN_METABOLISM	2.11825	0.00000	0.00061
KEGG_RETINOL_METABOLISM	2.07964	0.00000	0.00049
KEGG_STEROID_HORMONE_BIOSYNTHESIS	2.01538	0.00000	0.00193
KEGG_ABC_TRANSPORTERS	1.95824	0.00000	0.00341
KEGG_TYROSINE_METABOLISM	1.95700	0.00304	0.00298

KEGG_BUTANOATE_METABOLISM	1.93400	0.00000	0.00342
KEGG_PPAR_SIGNALING_PATHWAY	1.89790	0.00369	0.00457
KEGG_PRIMARY_BILE_ACID_BIOSYNTHESIS	1.83924	0.00264	0.00876
KEGG_OXIDATIVE_PHOSPHORYLATION	1.83895	0.00000	0.00803
KEGG_ASCORBATE_AND_ALDARATE_METABOLISM	1.82070	0.00535	0.00923
KEGG_PHENYLALANINE_METABOLISM	1.80087	0.00000	0.01081
KEGG_GLUTATHIONE_METABOLISM	1.78991	0.00000	0.01107
KEGG_PARKINSONS_DISEASE	1.78233	0.00000	0.01116
KEGG_STARCH_AND_SUCROSE_METABOLISM	1.73703	0.00322	0.01610
KEGG_VALINE_LEUCINE_AND_ISOLEUCINE_DEGRADATION	1.73233	0.00000	0.01674
KEGG_TERPENOID_BACKBONE_BIOSYNTHESIS	1.69084	0.00730	0.02429
KEGG_PROPANOATE_METABOLISM	1.69038	0.00637	0.02317
KEGG_GLYCOSAMINOGLYCAN_DEGRADATION	1.67024	0.01401	0.02647
KEGG_DRUG_METABOLISM_OTHER_ENZYMES	1.67010	0.00643	0.02527
KEGG_ARACHIDONIC_ACID_METABOLISM	1.66921	0.00360	0.02433
KEGG_LINOLEIC_ACID_METABOLISM	1.65254	0.01120	0.02698
KEGG_PORPHYRIN_AND_CHLOROPHYLL_METABOLISM	1.64448	0.01453	0.02752
KEGG_PEROXISOME	1.63287	0.00826	0.02866
KEGG_PROTEIN_EXPORT	1.62634	0.01724	0.02948
KEGG_FATTY_ACID_METABOLISM	1.61759	0.00697	0.03039
KEGG_CITRATE_CYCLE_TCA_CYCLE	1.58978	0.02360	0.03559
KEGG_HISTIDINE_METABOLISM	1.50369	0.02793	0.06586
KEGG_OTHER_GLYCAN_DEGRADATION	1.47624	0.06266	0.07697
KEGG_NICOTINATE_AND_NICOTINAMIDE_METABOLISM	1.46121	0.05121	0.08357
KEGG_PYRUVATE_METABOLISM	1.44074	0.02941	0.09333

NES: normalized enrichment score, nom p-value=nominal p-value, FDR q-value=false discovery rate p-value.

Supplementary Tables 8a-b. Multivariable Cox regression results and parameters for recurrent-free interval (RFI) (a) and breast cancer-specific survival (BCSS) (b) of the METABRIC cohort analysis comparing the patients with high and low expression of the top 5 up- and downregulated genes (except *TRAJ14*, Entrez ID: 28741, not present in the METABRIC analysis) and top 10 differentially expressed Hallmark gene sets, obtained from the analysis of primary tumors of endocrine-resistant and endocrine-sensitive patients in the Endoresist cohort.

a.

Characteristic	Univariate HR[1]	Univariate 95% CI[1]	Univariate p-value	Multivariable HR[1]	Multivariable 95% CI[1]	Multivariable p-value
**AGR2**						
scale(AGR2)	1.10	0.98, 1.23	0.093	1.06	0.94, 1.18	0.4
lnstat_						
LN-				—	—	
LN+				1.73	1.35, 2.21	<0.001
Unknown						
agecat_						
<50				—	—	
>=50				0.95	0.63, 1.43	0.8
Unknown						
tumsizecat_						
<=20mm				—	—	
>20mm				1.74	1.35, 2.24	<0.001
Unknown				0.59	0.08, 4.32	0.6
**RPN1**						
scale(RPN1)	1.19	1.07, 1.33	0.002	1.19	1.07, 1.33	0.002
lnstat_						
LN-				—	—	
LN+				1.76	1.38, 2.26	<0.001
Unknown						
agecat_						
<50				—	—	
>=50				0.98	0.65, 1.47	>0.9
Unknown						
tumsizecat_						
<=20mm				—	—	
>20mm				1.74	1.35, 2.23	<0.001
Unknown				0.63	0.09, 4.66	0.7
**RPL30**						
scale(RPL30)	1.00	0.90, 1.12	>0.9	1.01	0.91, 1.13	0.8
lnstat_						
LN-				—	—	
LN+				1.75	1.36, 2.23	<0.001
Unknown						
agecat_						
<50				—	—	

[1] HR = Hazard Ratio, CI = Confidence Interval

Characteristic	Univariate			Multivariable		
	HR[1]	95% CI[1]	p-value	HR[1]	95% CI[1]	p-value
>=50				0.96	0.64, 1.44	0.8
Unknown						
tumsizecat_						
<=20mm				—	—	
>20mm				1.75	1.36, 2.25	<0.001
Unknown				0.59	0.08, 4.37	0.6
**CD163**						
scale(CD163)	1.07	0.95, 1.21	0.3	1.10	0.97, 1.25	0.14
lnstat_						
LN-				—	—	
LN+				1.75	1.37, 2.23	<0.001
Unknown						
agecat_						
<50				—	—	
>=50				0.94	0.63, 1.42	0.8
Unknown						
tumsizecat_						
<=20mm				—	—	
>20mm				1.77	1.38, 2.28	<0.001
Unknown				0.63	0.09, 4.65	0.7
**ITGB1**						
scale(ITGB1)	1.02	0.91, 1.14	0.8	1.05	0.93, 1.18	0.4
lnstat_						
LN-				—	—	
LN+				1.75	1.37, 2.24	<0.001
Unknown						
agecat_						
<50				—	—	
>=50				0.97	0.65, 1.46	0.9
Unknown						
tumsizecat_						
<=20mm				—	—	
>20mm				1.76	1.37, 2.27	<0.001
Unknown				0.61	0.08, 4.53	0.6
**CLIC6**						
scale(CLIC6)	0.83	0.73, 0.93	0.001	0.84	0.75, 0.95	0.004
lnstat_						

[1] HR = Hazard Ratio, CI = Confidence Interval

Characteristic	Univariate HR[1]	Univariate 95% CI[1]	Univariate p-value	Multivariable HR[1]	Multivariable 95% CI[1]	Multivariable p-value
LN-				—	—	
LN+				1.75	1.37, 2.24	<0.001
Unknown						
agecat_						
<50				—	—	
>=50				0.90	0.60, 1.36	0.6
Unknown						
tumsizecat_						
<=20mm				—	—	
>20mm				1.70	1.32, 2.18	<0.001
Unknown				0.53	0.07, 3.88	0.5
**SLC39A6**						
scale(SLC39A6)	0.84	0.76, 0.94	0.003	0.82	0.74, 0.92	<0.001
lnstat_						
LN-				—	—	
LN+				1.77	1.38, 2.26	<0.001
Unknown						
agecat_						
<50				—	—	
>=50				0.98	0.65, 1.46	>0.9
Unknown						
tumsizecat_						
<=20mm				—	—	
>20mm				1.79	1.40, 2.30	<0.001
Unknown				0.52	0.07, 3.77	0.5
**VTCN1**						
scale(VTCN1)	0.86	0.77, 0.96	0.006	0.86	0.77, 0.96	0.009
lnstat_						
LN-				—	—	
LN+				1.76	1.38, 2.25	<0.001
Unknown						
agecat_						
<50				—	—	
>=50				0.92	0.61, 1.39	0.7
Unknown						
tumsizecat_						
<=20mm				—	—	

[1] HR = Hazard Ratio, CI = Confidence Interval

Characteristic	Univariate HR[1]	Univariate 95% CI[1]	Univariate p-value	Multivariable HR[1]	Multivariable 95% CI[1]	Multivariable p-value
>20mm				1.72	1.34, 2.21	<0.001
Unknown				0.59	0.08, 4.35	0.6
**ZNF385B**						
scale(ZNF385B)	0.90	0.80, 1.01	0.067	0.91	0.81, 1.02	0.10
lnstat_						
LN-				—	—	
LN+				1.75	1.37, 2.23	<0.001
Unknown						
agecat_						
<50				—	—	
>=50				0.95	0.63, 1.43	0.8
Unknown						
tumsizecat_						
<=20mm				—	—	
>20mm				1.73	1.35, 2.22	<0.001
Unknown				0.56	0.08, 4.09	0.6
**HALLMARK_E2F_TARGETS**						
scale(HALLMARK_E2F_TARGETS)	1.33	1.19, 1.49	<0.001	1.31	1.17, 1.47	<0.001
lnstat_						
LN-				—	—	
LN+				1.79	1.40, 2.29	<0.001
Unknown						
agecat_						
<50				—	—	
>=50				0.99	0.66, 1.48	>0.9
Unknown						
tumsizecat_						
<=20mm				—	—	
>20mm				1.63	1.27, 2.10	<0.001
Unknown				0.52	0.07, 3.79	0.5
**HALLMARK_G2M_CHECKPOINT**						
scale(HALLMARK_G2M_CHECKPOINT)	1.43	1.27, 1.61	<0.001	1.41	1.26, 1.59	<0.001
lnstat_						
LN-				—	—	
LN+				1.80	1.40, 2.30	<0.001
Unknown						
agecat_						

[1] HR = Hazard Ratio, CI = Confidence Interval

Characteristic	Univariate			Multivariable		
	HR[1]	95% CI[1]	p-value	HR[1]	95% CI[1]	p-value
<50				—	—	
>=50				1.00	0.67, 1.51	>0.9
Unknown						
tumsizecat_						
<=20mm				—	—	
>20mm				1.62	1.26, 2.08	<0.001
Unknown				0.50	0.07, 3.68	0.5
**HALLMARK_DNA_REPAIR**						
scale(HALLMARK_DNA_REPAIR)	1.26	1.12, 1.42	<0.001	1.24	1.10, 1.39	<0.001
lnstat_						
LN-				—	—	
LN+				1.76	1.38, 2.25	<0.001
Unknown						
agecat_						
<50				—	—	
>=50				0.98	0.65, 1.47	>0.9
Unknown						
tumsizecat_						
<=20mm				—	—	
>20mm				1.67	1.30, 2.15	<0.001
Unknown				0.54	0.07, 3.94	0.5
**HALLMARK_UNFOLDED_PROTEIN_RESPONSE**						
scale(HALLMARK_UNFOLDED_PROTEIN_RESPONSE)	1.21	1.08, 1.36	<0.001	1.19	1.06, 1.33	0.003
lnstat_						
LN-				—	—	
LN+				1.77	1.38, 2.26	<0.001
Unknown						
agecat_						
<50				—	—	
>=50				0.99	0.66, 1.49	>0.9
Unknown						
tumsizecat_						
<=20mm				—	—	
>20mm				1.68	1.30, 2.16	<0.001
Unknown				0.54	0.07, 3.99	0.5
**HALLMARK_MYC_TARGETS_V1**						
scale(HALLMARK_MYC_TARGETS_V1)	1.24	1.10, 1.39	<0.001	1.22	1.08, 1.37	<0.001

[1] HR = Hazard Ratio, CI = Confidence Interval

	Univariate			Multivariable		
**Characteristic**	**HR**[1]	**95% CI**[1]	**p-value**	**HR**[1]	**95% CI**[1]	**p-value**
lnstat_						
LN-				—	—	
LN+				1.77	1.38, 2.27	<0.001
Unknown						
agecat_						
<50				—	—	
>=50				1.02	0.68, 1.53	>0.9
Unknown						
tumsizecat_						
<=20mm				—	—	
>20mm				1.67	1.30, 2.15	<0.001
Unknown				0.57	0.08, 4.18	0.6
**HALLMARK_GLYCOLYSIS**						
scale(HALLMARK_GLYCOLYSIS)	1.15	1.02, 1.29	0.019	1.13	1.00, 1.27	0.045
lnstat_						
LN-				—	—	
LN+				1.76	1.38, 2.25	<0.001
Unknown						
agecat_						
<50				—	—	
>=50				0.98	0.65, 1.47	>0.9
Unknown						
tumsizecat_						
<=20mm				—	—	
>20mm				1.70	1.32, 2.19	<0.001
Unknown				0.60	0.08, 4.44	0.6
**HALLMARK_MITOTIC_SPINDLE**						
scale(HALLMARK_MITOTIC_SPINDLE)	1.36	1.20, 1.55	<0.001	1.39	1.22, 1.58	<0.001
lnstat_						
LN-				—	—	
LN+				1.82	1.42, 2.33	<0.001
Unknown						
agecat_						
<50				—	—	
>=50				1.00	0.67, 1.51	>0.9
Unknown						
tumsizecat_						

[1] HR = Hazard Ratio, CI = Confidence Interval

Characteristic	Univariate			Multivariable		
	HR[1]	95% CI[1]	p-value	HR[1]	95% CI[1]	p-value
<=20mm				—	—	
>20mm				1.69	1.32, 2.18	<0.001
Unknown				0.57	0.08, 4.22	0.6
**HALLMARK_HEME_METABOLISM**						
scale(HALLMARK_HEME_METABOLISM)	0.89	0.79, 1.00	0.048	0.90	0.81, 1.01	0.087
lnstat_						
LN-				—	—	
LN+				1.74	1.36, 2.23	<0.001
Unknown						
agecat_						
<50				—	—	
>=50				0.98	0.65, 1.48	>0.9
Unknown						
tumsizecat_						
<=20mm				—	—	
>20mm				1.73	1.35, 2.23	<0.001
Unknown				0.52	0.07, 3.87	0.5
**HALLMARK_MTORC1_SIGNALING**						
scale(HALLMARK_MTORC1_SIGNALING)	1.28	1.14, 1.44	<0.001	1.26	1.12, 1.41	<0.001
lnstat_						
LN-				—	—	
LN+				1.76	1.38, 2.25	<0.001
Unknown						
agecat_						
<50				—	—	
>=50				0.99	0.66, 1.48	>0.9
Unknown						
tumsizecat_						
<=20mm				—	—	
>20mm				1.68	1.30, 2.16	<0.001
Unknown				0.56	0.08, 4.10	0.6
**HALLMARK_INTERFERON_GAMMA_RESPONSE**						
scale(HALLMARK_INTERFERON_GAMMA_RESPONSE)	1.08	0.97, 1.21	0.2	1.11	0.99, 1.25	0.068
lnstat_						
LN-				—	—	
LN+				1.74	1.36, 2.22	<0.001
Unknown						

[1] HR = Hazard Ratio, CI = Confidence Interval

Characteristic	Univariate			Multivariable		
	HR[1]	95% CI[1]	p-value	HR[1]	95% CI[1]	p-value
agecat_						
<50				—	—	
>=50				0.95	0.63, 1.43	0.8
Unknown						
tumsizecat_						
<=20mm				—	—	
>20mm				1.79	1.39, 2.30	<0.001
Unknown				0.57	0.08, 4.21	0.6

[1] HR = Hazard Ratio, CI = Confidence Interval

b.

Characteristic	Univariate			Multivariable		
	HR[1]	95% CI[1]	p-value	HR[1]	95% CI[1]	p-value
**AGR2**						
scale(AGR2)	1.14	1.00, 1.31	0.047	1.08	0.94, 1.23	0.3
lnstat_						
LN-				—	—	
LN+				2.02	1.49, 2.74	<0.001
Unknown						
agecat_						
<50				—	—	
>=50				2.11	1.11, 4.02	0.023
Unknown						
tumsizecat_						
<=20mm				—	—	
>20mm				1.83	1.35, 2.49	<0.001
Unknown				0.00	0.00, Inf	>0.9
**RPN1**						
scale(RPN1)	1.18	1.03, 1.35	0.015	1.17	1.03, 1.33	0.018
lnstat_						
LN-				—	—	
LN+				2.06	1.52, 2.79	<0.001
Unknown						
agecat_						
<50				—	—	
>=50				2.17	1.14, 4.13	0.019
Unknown						
tumsizecat_						
<=20mm				—	—	
>20mm				1.83	1.35, 2.48	<0.001
Unknown				0.00	0.00, Inf	>0.9
**RPL30**						
scale(RPL30)	0.96	0.84, 1.09	0.5	0.96	0.85, 1.10	0.6
lnstat_						
LN-				—	—	
LN+				2.04	1.50, 2.76	<0.001
Unknown						
agecat_						
<50				—	—	

[1] HR = Hazard Ratio, CI = Confidence Interval

Characteristic	Univariate			Multivariable		
	HR[1]	95% CI[1]	p-value	HR[1]	95% CI[1]	p-value
>=50				2.15	1.13, 4.09	0.020
Unknown						
tumsizecat_						
<=20mm				—	—	
>20mm				1.86	1.37, 2.52	<0.001
Unknown				0.00	0.00, Inf	>0.9
**CD163**						
scale(CD163)	1.26	1.09, 1.45	0.002	1.28	1.11, 1.49	<0.001
lnstat_						
LN-				—	—	
LN+				2.08	1.53, 2.82	<0.001
Unknown						
agecat_						
<50				—	—	
>=50				2.01	1.06, 3.84	0.034
Unknown						
tumsizecat_						
<=20mm				—	—	
>20mm				1.92	1.42, 2.60	<0.001
Unknown				0.00	0.00, Inf	>0.9
**ITGB1**						
scale(ITGB1)	0.99	0.86, 1.14	0.9	1.03	0.90, 1.19	0.6
lnstat_						
LN-				—	—	
LN+				2.05	1.52, 2.78	<0.001
Unknown						
agecat_						
<50				—	—	
>=50				2.16	1.13, 4.11	0.019
Unknown						
tumsizecat_						
<=20mm				—	—	
>20mm				1.86	1.38, 2.52	<0.001
Unknown				0.00	0.00, Inf	>0.9
**CLIC6**						
scale(CLIC6)	0.75	0.65, 0.86	<0.001	0.78	0.68, 0.90	<0.001
lnstat_						

[1] HR = Hazard Ratio, CI = Confidence Interval

Characteristic	Univariate			Multivariable		
	HR[1]	95% CI[1]	p-value	HR[1]	95% CI[1]	p-value
LN-				—	—	
LN+				2.06	1.52, 2.79	<0.001
Unknown						
agecat_						
<50				—	—	
>=50				1.98	1.04, 3.77	0.038
Unknown						
tumsizecat_						
<=20mm				—	—	
>20mm				1.78	1.31, 2.41	<0.001
Unknown				0.00	0.00, Inf	>0.9
**SLC39A6**						
scale(SLC39A6)	0.83	0.73, 0.95	0.007	0.80	0.70, 0.91	<0.001
lnstat_						
LN-				—	—	
LN+				2.08	1.54, 2.82	<0.001
Unknown						
agecat_						
<50				—	—	
>=50				2.20	1.15, 4.18	0.017
Unknown						
tumsizecat_						
<=20mm				—	—	
>20mm				1.91	1.41, 2.58	<0.001
Unknown				0.00	0.00, Inf	>0.9
**VTCN1**						
scale(VTCN1)	0.80	0.70, 0.91	<0.001	0.81	0.71, 0.93	0.002
lnstat_						
LN-				—	—	
LN+				2.09	1.54, 2.83	<0.001
Unknown						
agecat_						
<50				—	—	
>=50				2.05	1.07, 3.90	0.030
Unknown						
tumsizecat_						
<=20mm				—	—	

[1] HR = Hazard Ratio, CI = Confidence Interval

Characteristic	Univariate			Multivariable		
	HR[1]	95% CI[1]	p-value	HR[1]	95% CI[1]	p-value
>20mm				1.80	1.33, 2.44	<0.001
Unknown				0.00	0.00, Inf	>0.9
**ZNF385B**						
scale(ZNF385B)	0.85	0.73, 0.98	0.022	0.87	0.75, 1.00	0.056
lnstat_						
LN-				—	—	
LN+				2.04	1.51, 2.77	<0.001
Unknown						
agecat_						
<50				—	—	
>=50				2.13	1.12, 4.06	0.021
Unknown						
tumsizecat_						
<=20mm				—	—	
>20mm				1.81	1.34, 2.46	<0.001
Unknown				0.00	0.00, Inf	>0.9
**HALLMARK_E2F_TARGETS**						
scale(HALLMARK_E2F_TARGETS)	1.40	1.22, 1.60	<0.001	1.37	1.20, 1.58	<0.001
lnstat_						
LN-				—	—	
LN+				2.10	1.55, 2.85	<0.001
Unknown						
agecat_						
<50				—	—	
>=50				2.20	1.15, 4.19	0.017
Unknown						
tumsizecat_						
<=20mm				—	—	
>20mm				1.68	1.23, 2.28	<0.001
Unknown				0.00	0.00, Inf	>0.9
**HALLMARK_G2M_CHECKPOINT**						
scale(HALLMARK_G2M_CHECKPOINT)	1.49	1.30, 1.71	<0.001	1.47	1.28, 1.69	<0.001
lnstat_						
LN-				—	—	
LN+				2.11	1.56, 2.86	<0.001
Unknown						
agecat_						

[1] HR = Hazard Ratio, CI = Confidence Interval

Characteristic	Univariate			Multivariable		
	HR[1]	95% CI[1]	p-value	HR[1]	95% CI[1]	p-value
<50				—	—	
>=50				2.23	1.17, 4.26	0.015
Unknown						
tumsizecat_						
<=20mm				—	—	
>20mm				1.66	1.22, 2.26	0.001
Unknown				0.00	0.00, Inf	>0.9
**HALLMARK_DNA_REPAIR**						
scale(HALLMARK_DNA_REPAIR)	1.31	1.14, 1.51	<0.001	1.28	1.11, 1.48	<0.001
lnstat_						
LN-				—	—	
LN+				2.06	1.52, 2.79	<0.001
Unknown						
agecat_						
<50				—	—	
>=50				2.17	1.14, 4.14	0.018
Unknown						
tumsizecat_						
<=20mm				—	—	
>20mm				1.73	1.27, 2.35	<0.001
Unknown				0.00	0.00, Inf	>0.9
**HALLMARK_UNFOLDED_PROTEIN_RESPONSE**						
scale(HALLMARK_UNFOLDED_PROTEIN_RESPONSE)	1.22	1.06, 1.40	0.004	1.20	1.05, 1.37	0.009
lnstat_						
LN-				—	—	
LN+				2.07	1.52, 2.80	<0.001
Unknown						
agecat_						
<50				—	—	
>=50				2.20	1.16, 4.19	0.016
Unknown						
tumsizecat_						
<=20mm				—	—	
>20mm				1.77	1.30, 2.40	<0.001
Unknown				0.00	0.00, Inf	>0.9
**HALLMARK_MYC_TARGETS_V1**						
scale(HALLMARK_MYC_TARGETS_V1)	1.19	1.04, 1.37	0.012	1.18	1.03, 1.36	0.018

[1] HR = Hazard Ratio, CI = Confidence Interval

Characteristic	Univariate			Multivariable		
	HR[1]	95% CI[1]	p-value	HR[1]	95% CI[1]	p-value
lnstat_						
LN-				—	—	
LN+				2.07	1.53, 2.80	<0.001
Unknown						
agecat_						
<50				—	—	
>=50				2.25	1.18, 4.28	0.014
Unknown						
tumsizecat_						
<=20mm				—	—	
>20mm				1.77	1.30, 2.40	<0.001
Unknown				0.00	0.00, Inf	>0.9
**HALLMARK_GLYCOLYSIS**						
scale(HALLMARK_GLYCOLYSIS)	1.19	1.03, 1.37	0.015	1.17	1.01, 1.35	0.032
lnstat_						
LN-				—	—	
LN+				2.07	1.53, 2.80	<0.001
Unknown						
agecat_						
<50				—	—	
>=50				2.18	1.15, 4.15	0.018
Unknown						
tumsizecat_						
<=20mm				—	—	
>20mm				1.78	1.31, 2.42	<0.001
Unknown				0.00	0.00, Inf	>0.9
**HALLMARK_MITOTIC_SPINDLE**						
scale(HALLMARK_MITOTIC_SPINDLE)	1.52	1.30, 1.77	<0.001	1.55	1.33, 1.81	<0.001
lnstat_						
LN-				—	—	
LN+				2.16	1.59, 2.93	<0.001
Unknown						
agecat_						
<50				—	—	
>=50				2.23	1.17, 4.25	0.014
Unknown						
tumsizecat_						

[1] HR = Hazard Ratio, CI = Confidence Interval

Characteristic	Univariate HR[1]	Univariate 95% CI[1]	Univariate p-value	Multivariable HR[1]	Multivariable 95% CI[1]	Multivariable p-value
<=20mm				—	—	
>20mm				1.74	1.28, 2.36	<0.001
Unknown				0.00	0.00, Inf	>0.9
**HALLMARK_HEME_METABOLISM**						
scale(HALLMARK_HEME_METABOLISM)	0.89	0.78, 1.02	0.10	0.89	0.78, 1.03	0.11
lnstat_						
LN-				—	—	
LN+				2.05	1.51, 2.77	<0.001
Unknown						
agecat_						
<50				—	—	
>=50				2.20	1.15, 4.20	0.017
Unknown						
tumsizecat_						
<=20mm				—	—	
>20mm				1.83	1.35, 2.47	<0.001
Unknown				0.00	0.00, Inf	>0.9
**HALLMARK_MTORC1_SIGNALING**						
scale(HALLMARK_MTORC1_SIGNALING)	1.37	1.19, 1.57	<0.001	1.34	1.17, 1.55	<0.001
lnstat_						
LN-				—	—	
LN+				2.07	1.53, 2.80	<0.001
Unknown						
agecat_						
<50				—	—	
>=50				2.21	1.16, 4.21	0.016
Unknown						
tumsizecat_						
<=20mm				—	—	
>20mm				1.75	1.29, 2.37	<0.001
Unknown				0.00	0.00, Inf	>0.9
**HALLMARK_INTERFERON_GAMMA_RESPONSE**						
scale(HALLMARK_INTERFERON_GAMMA_RESPONSE)	1.17	1.03, 1.34	0.018	1.21	1.06, 1.39	0.006
lnstat_						
LN-				—	—	
LN+				2.04	1.51, 2.77	<0.001
Unknown						

[1] HR = Hazard Ratio, CI = Confidence Interval

	Univariate			Multivariable		
**Characteristic**	**HR**[1]	**95% CI**[1]	**p-value**	**HR**[1]	**95% CI**[1]	**p-value**
agecat_						
<50				—	—	
>=50				2.10	1.10, 4.00	0.024
Unknown						
tumsizecat_						
<=20mm				—	—	
>20mm				1.94	1.43, 2.62	<0.001
Unknown				0.00	0.00, Inf	>0.9

[1] HR = Hazard Ratio, CI = Confidence Interval
